# Tumor-initiating cells escape tumor immunity via CCL8 from tumor-associated macrophages in mice

**DOI:** 10.1172/JCI180893

**Published:** 2025-01-07

**Authors:** Shuang Chen, Chensong Huang, Kang Li, Maosheng Cheng, Caihua Zhang, Jianqi Xiong, Guoli Tian, Ruoxing Zhou, Rongsong Ling, Xiaochen Wang, Gan Xiong, Zhihui Zhang, Jieyi Ma, Yan Zhu, Bin Zhou, Liang Peng, Zhenwei Peng, Heping Li, Demeng Chen

**Affiliations:** 1 Department of Otorhinolaryngology, Department of Medical Oncology, Department of Pancreato-Biliary Surgery, Department of Radiation Oncology, Cancer Center, Center for Translational Medicine, The First Affiliated Hospital of Sun Yat-sen University (FAHSYSU), Guangzhou, China.; 2 Hospital of Stomatology, Sun Yat-sen University, Guangzhou, China.; 3 Department of Medical Oncology, Sun Yat-sen Memorial Hospital, Sun Yat-sen University, Guangzhou, China.; 4 State Key Laboratory of Cell Biology, Shanghai Institute of Biochemistry and Cell Biology, Center for Excellence in Molecular Cell Science, Chinese Academy of Sciences, University of Chinese Academy of Sciences, Shanghai, China.; 5 Senior Department of Oncology, The Fifth Medical Center of PLA General Hospital, Beijing, China.; 6 State Key Laboratory of Oncology in South China, Sun Yat-sen University Cancer Center, Guangzhou, China.

**Keywords:** Oncology, Cancer immunotherapy

## Abstract

Tumor-initiating cells (TICs) play a key role in cancer progression and immune escape. However, how TICs evade immune elimination remains poorly characterized. Combining single-cell RNA-Seq (scRNA-Seq), dual-recombinase–based lineage tracing, and other approaches, we identified a WNT-activated subpopulation of malignant cells that act as TICs in vivo. We found intensive reciprocal interactions between TICs and immune-regulatory tumor-associated macrophages (Reg-TAMs) via growth arrest–specific 6/AXL receptor tyrosine kinase/MER proto-oncogene, tyrosine kinase (GAS6/AXL/MERTK) signaling pathways, which facilitated the immune escape of TICs. In this study, we used chemical inhibitors and *Axl*/*Mertk* conditional double-KO (cDKO) mice to demonstrate that inhibiting the interaction between TIC-derived GAS6 and AXL/MERTK in Reg-TAMs reactivated antitumor immune responses. We identified CCL8 as a critical mediator of the GAS6/AXL/MERTK pathway, primarily by inhibiting Treg infiltration into the tumor. Furthermore, the AXL/MERTK signaling blockade sensitized tumor cells to anti–programmed cell death 1 (anti–PD-1) treatment. Thus, we elucidated a detailed mechanism by which TICs evade tumor immunity, providing insights into strategies to eradicate TICs that escape conventional immunotherapy.

## Abstract 2

We identifies WNT-activated tumor-initiating cells evading immune attacks by interacting with macrophages through the GAS6/AXL/MERTK pathway, suggesting new targets for cancer therapy.

## Introduction

As the second most common primary liver cancer, intrahepatic cholangiocarcinoma (ICC) is highly aggressive with a poor prognosis. The majority of patients with ICC (>70%) are already at advanced stages at the time of diagnosis and cannot be surgically treated due to locally advanced or metastatic disease (1, 2). Thus far, effective therapeutic strategies for patients with ICC are limited. In addition, the highly heterogeneous nature of ICC at both genetic and phenotypic levels often leads to treatment failure and drug resistance.

Evidence suggests that tumor-initiating cells (TICs), a rare self-renewing population with multilineage differentiation potential and immune evasive properties, drive tumor heterogeneity (3). Historically, TICs in ICC have been characterized by markers such as KIT, SALL4, CD44, and SOX2 (4). However, traditional approaches to identifying TICs often disrupt their architecture and interactions with the tumor microenvironment (TME). In addition, evidence shows that immune evasion is a key feature distinguishing TICs from non-TICs (5), but how TICs in ICC evade immune surveillance and how they shape an immunosuppressive TME remains unclear. Therefore, elucidating this mechanism and the immunological characteristics of TICs in ICC will improve our understanding of ICC development. To do so, it is crucial to pinpoint the TIC population within their niche in vivo. Lineage tracing has become a well-suited approach for delineating the tumor cell of origin and tumor heterogeneity in mouse models (6). Moreover, the fast-advancing technique of single-cell RNA-Seq (scRNA-Seq) has allowed us to gain deeper insights into tumor heterogeneity and the interplay within the tumor ecosystem (7).

In this study, we performed scRNA-Seq to explore the tumor heterogeneity of murine ICC. We identified 18 cell types on the basis of signature genes, categorized into 6 major groups: epithelial, stromal, NKT cells, B cells, monocyte/macrophage/DCs (MoMϕDCs), and neutrophils in murine ICC. These findings are highly consistent with online patient ICC scRNA-Seq data (8). Further analysis revealed that a WNT-activated subcluster of malignant epithelial cells could potentially give rise to other subclusters. We used a dual-recombinase–based lineage-tracing system to verify these findings and track WNT-activated epithelial cells (AXIN2^+^KRT19^+^) in the mouse ICC model. We found that these cells were responsible for ICC development and progression. We then constructed a cell-cell interaction network based on scRNA-Seq data. We found that WNT-activated malignant epithelial cells interacted intensively with immune-regulatory tumor-associated macrophages (Reg-TAMs) via the growth arrest–specific 6/AXL receptor tyrosine kinase/MER proto-oncogene, tyrosine kinase (GAS6/AXL/MERTK) pathway. Functionally, inhibition of the GAS6/AXL/MERTK pathway led to repression of CCL8 expression in Reg-TAMs and impaired stemness of TICs in mouse ICC. Finally, we observed a synergistic effect of an AXL/MERTK inhibitor with anti–PD-1 treatment on ICC. These results provide a rationale for the combination of an AXL/MERTK inhibitor and anti–PD-1 antibody in ICC, and these findings might ultimately help identify a more effective combination regimen to elicit stronger antitumor responses.

## Results

### Single-cell sequencing and cell-type identification in murine ICC.

**Figure 1:**
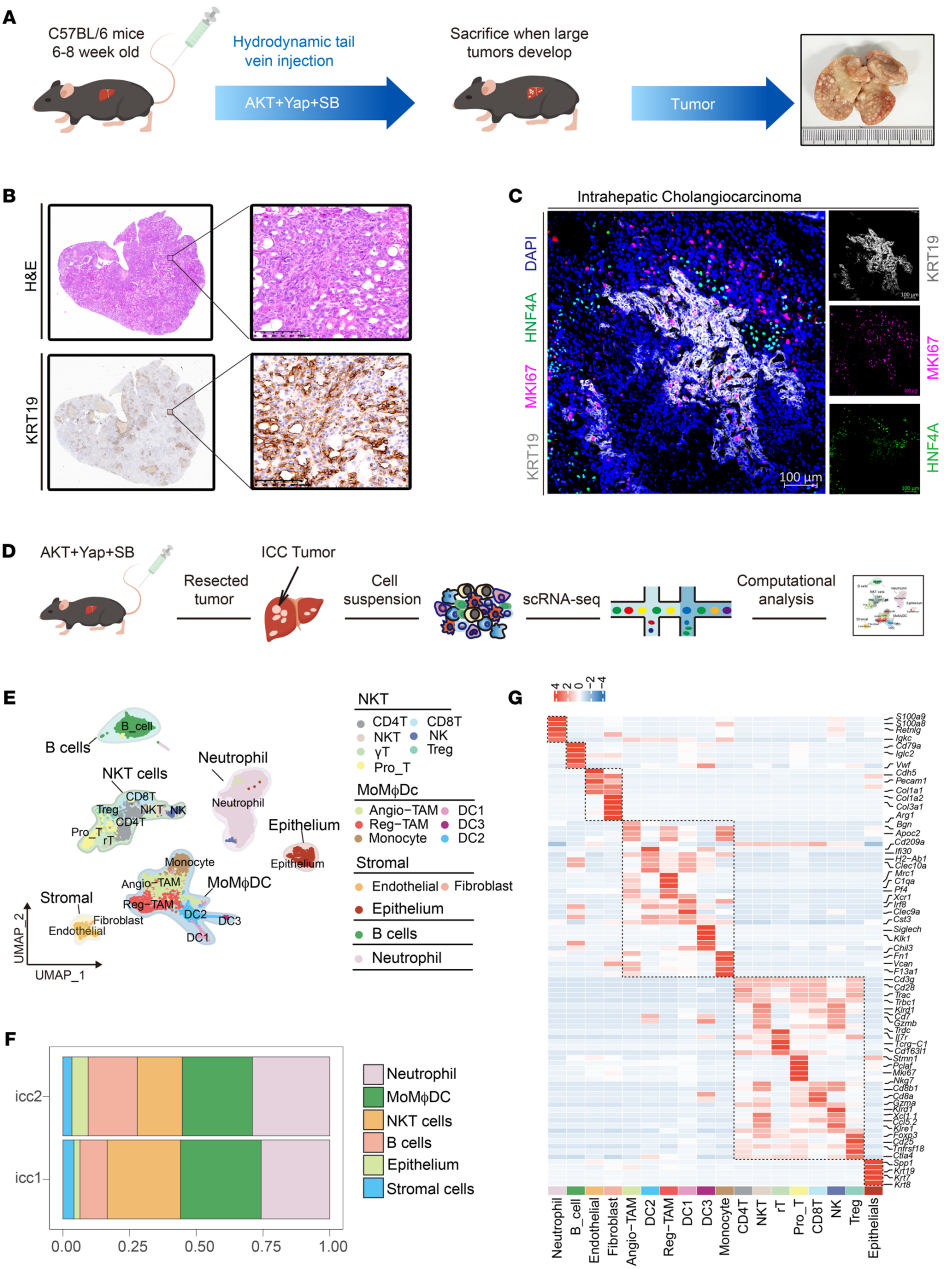
Heterogeneity of cells in mouse ICC sample. (**A**) Schematic of mouse ICC induction workflow. Mice were injected with AKT/YAP/SB plasmid via the tail vein, and tumor-bearing mice were sacrificed when large tumors developed (*n* = 3). (**B**) Representative images of H&E and KRT19 IHC staining of liver sections from ICC mice (*n* = 3 mice). Scale bars: 100 μm. (**C**) Opal/TSA multicolor IF staining for anti-KRT19, anti-MKI67, and anti-HNF4A antibodies; nuclei are stained with DAPI (blue) (*n* = 3 mice). Scale bar: 100 μm. (**D**) Scheme of the workflow for ICC cell isolation and single-cell RNA-Seq. (**E**) UMAP of single-cell clusters from mouse ICC tumor tissues (*n* = 2), colored by cluster. (**F**) Proportions of single-cell clusters in each sample. (**G**) Heatmap of signature genes for 18 cell clusters.

To investigate the tumor ecosystem and molecular signature in ICC, we performed hydrodynamic tail vein injection of HA-tagged AKT serine/threonine kinase (AKT) and YAP-S127A (plasmid encoding a mutant form of YAP with a mutation at S127A with Sleeping Beauty (SB) plasmids into 6- to 8-week-old C57BL/6 mice (WT) (9, 10) (Figure 1A). We detected multiple lesions of various sizes in mouse livers 6 weeks after injection (Figure 1, A and B). Three independent pathologists from the FAHSYSU reviewed H&E, keratin 19 (KRT19) IHC, and immunofluorescence (IF) slides. These lesions were validated as cholangiocellular by positive KRT19 and negative HNF4A staining (Figure 1, B and C). Additionally, the AKT/YAP-induced ICC exhibited high proliferation marked by MKI67 (Figure 1C).

Subsequently, we dissected the ICC lesions macroscopically for the generation of scRNA-Seq profiles from 2 mice using 10× Genomics Sequencing (Figure 1D). After quality control and unsupervised clustering based on differentially expressed genes, we identified 18 cell types, categorized into 6 major groups: epithelium (*Krt19*, *Krt7*, *Krt8*), stromal (*Cdh5*, *Pecam1*, *Col1a2*), neutrophils (*S100a9*, *S100a8*, *Retnlg*), MoMϕDCs (*Csfr1*, *C1qa*, *Vcan*, *H2-Ab1*), NKT cells (*Cd3d*, *Gzmb*, *Klrd1*), and B cells (*Cd79a*, *Igkc*, *Iglc2*) (Figure 1, E and G, and Supplemental Figure 1A; supplemental material available online with this article; https://doi.org/10.1172/JCI180893DS1). We observed a similar distribution in both mouse ICC samples (Figure 1F).

Next, we compared murine scRNA-Seq data with a human ICC dataset of 14 samples(8). Six major cell clusters matched between datasets, showing high consistency and confirming the datasets’ quality (Supplemental Figure 1, B–F).

### WNT-activated cells act as TICs in mouse ICC.

**Figure 2:**
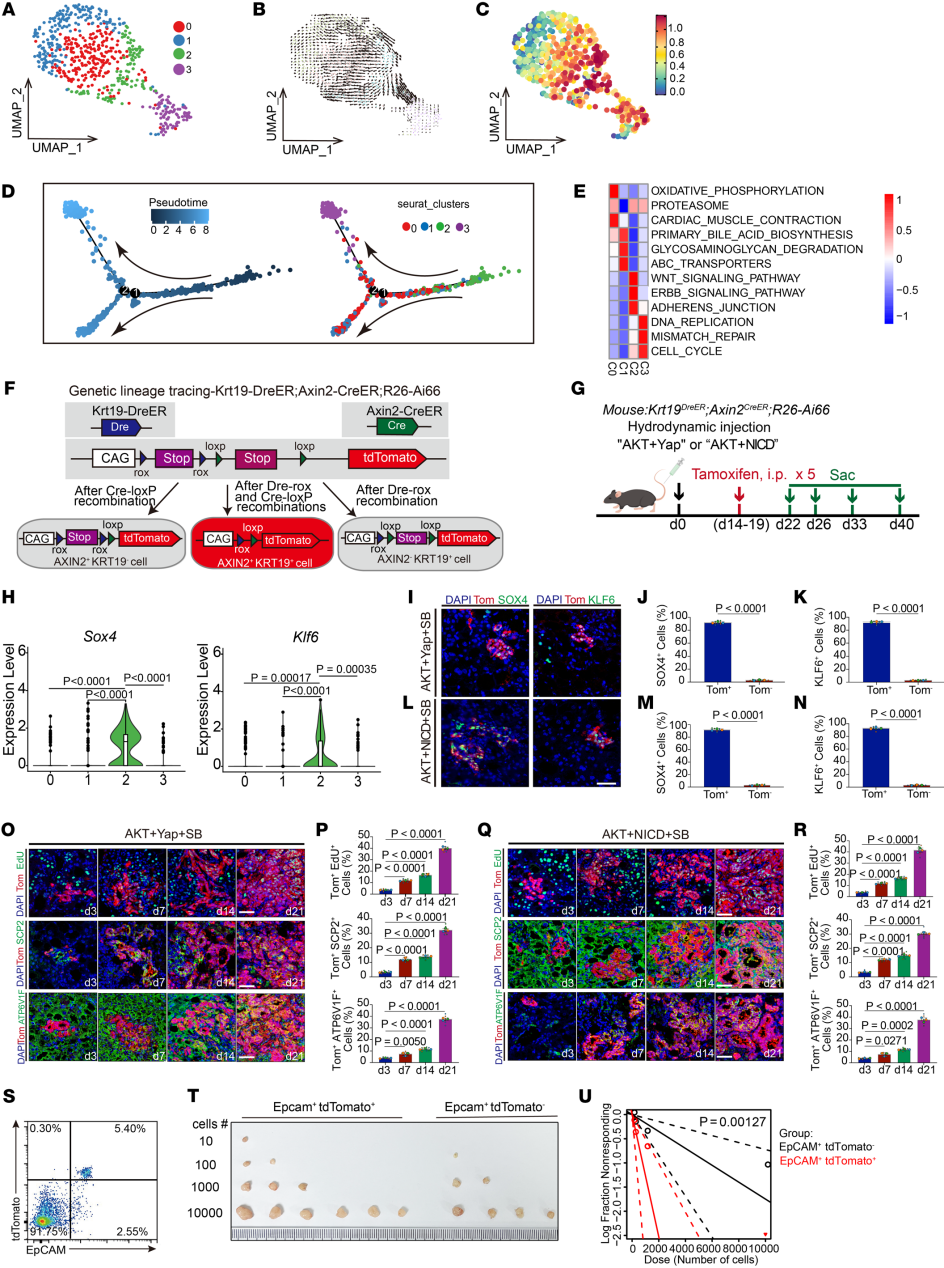
WNT-activated cells constitute a TIC population in mouse ICC. (**A**) UMAP plot showing 4 epithelial cell subclusters. (**B**) RNA velocity–inferred developmental trajectory of epithelial cells. (**C**) UMAP plots showing the distribution of CytoTRACE scores for epithelial cells. Higher scores indicate higher stemness. (**D**) Monocle pseudotime trajectory showing cell differentiation of 4 epithelial cell subclusters. (**E**) Heatmap of GSVA-enriched pathways in epithelial cell clusters. (**F**) Schematic of lineage tracing. (**G**) Experimental strategies for lineage tracing of AXIN2^+^KRT19^+^ epithelial cells in ICC mice. d0, day 0. (**H**) Violin plots showing *Sox4* and *Klf6* expression among epithelial cell subclusters. (**I** and **L**) Representative fluorescence images of SOX4 (green) and KLF6 (green) staining and Tom^+^ cells (red) in ICC tumors. Nuclei are stained with DAPI (blue). Scale bar: 50 μm. (**J**, **K**, **M**, and **N**) Statistical analysis of SOX4^+^ and KLF6^+^ expression in Tom^+/–^ mouse ICC cells. (**O** and **Q**) Representative fluorescence images showing EdU^+^ (green), SCP2^+^ (green), ATP6V1F^+^ (green), and Tom^+^ (red) cells in ICC after tamoxifen treatment. Nuclei are stained with DAPI (blue). Scale bars: 50 μm. (**P** and **R**) Comparison of the percentage of Tom^+^EdU^+^, Tom^+^SCP2^+^, and Tom^+^ATP6V1F^+^ cells in ICC induced for 3 days, with results shown at 7, 14, and 21 days. (**S**) Flow cytometry plots of EpCAM^+^Tom^+^ tumor cells that were isolated and from ICC tissues and sorted. (**T** and **U**) Tumor formation frequency of EpCAM^+^Tom^+^ and EpCAM^+^Tom^–^ ICC cells in vivo (**T**), analyzed by the single-hit model likelihood ratio test (**U**). Data represent the mean ± SD **J**, **K**, **P** and **R**). *P* values were calculated by 2-tailed, unpaired Student’s *t* test (**H**, **J**, **K**, **M**, and **N**) and 1-way ANOVA with Tukey’s multiple-comparison test (**P** and **R**).

To study the mechanisms regulating ICC development, we reclustered murine epithelial cells into 4 subclusters, C0–C3 (Figure 2A). We then used 3 trajectory inference analyses to measure transcriptional dynamics and characterize the differentiation process of these epithelial cell subclusters. First, RNA velocity analysis showed that the epithelial C2 subcluster gave rise to both C0 and C3 subclusters and further to C1 via C0 (Figure 2B). Second, CytoTRACE analysis revealed that C2 cells were more undifferentiated/less differentiated (Figure 2C). Lastly, pseudotemporal analysis (Monocle2) indicated that C2 cells exhibit higher progenitor properties and give rise to other epithelial clusters (Figure 2D). This suggests that C2 is a progenitor population that may give rise to other cell types. Further pathway analysis with gene set variation analysis (GSVA) based on Kyoto Encyclopedia of Genes and Genomes (KEGG) pathways demonstrated diverse molecular subtypes, with C2 enriched in WNT signaling, key pathways for stemness maintenance (Figure 2E) (11). Moreover, C2 showed higher WNT pathway scores compared with other subclusters, as assessed using the WNT signaling pathway gene set from the C2 modules of the Molecular Signatures Database (MSigDB) (Supplemental Figure 2A). Interestingly, subcluster C3, not C2, was enriched in DNA replication, mismatch repair, and cell-cycle pathways, with higher cell-cycle scores (Figure 2E and Supplemental Figure 2B), indicating that the C2 cells were not actively proliferating.

To investigate whether our finding of TICs in mouse models could faithfully recapitulate human ICC, we performed CytoTRACE analysis of malignant epithelial cells from each patient sample (Supplemental Figure 2C). Our results showed that epithelial cells with higher CytoTRACE scores tended to express higher levels of *CTNNB1* (Supplemental Figure 2C). Furthermore, correlation analysis using cholangiocarcinoma datasets from The Cancer Genome Atlas (TCGA-CHOL) showed that WNT pathway genes (*AXIN2*, *CTNNB1*, *LEF1*) positively correlated with TIC signature genes (*KIT*, *SALL4*, *CD44*, *SOX2*) (Supplemental Figure 2, D–G).

To test whether WNT-activated epithelial cells could act as TICs in ICC, we generated a dual-recombinase–based lineage-tracing system (*Krt19-DreER Axin2-CreER R26-Ai66*) mouse model. This model labels KRT19^+^AXIN2^+^ cells with tdTomato (Tom) upon tamoxifen administration, enabling the tracking of KRT19^+^AXIN2^+^ (Tom^+^) cell dynamics during ICC tumorigenesis (Figure 2F). Additionally, we conducted a time-course experiment to track Tom^+^ cells following hydrodynamic injection and observed the appearance of Tom^+^ cells 2 weeks after injection (Supplemental Figure 3, A–C). We then continued to monitor the dynamics of Tom^+^ cells during ICC progression. Two weeks after hydrodynamic injection, tamoxifen was administered to the mice (Figure 2G). From day 3 to week 3, after tamoxifen injection, we observed rapid expansion and progression of Tom^+^ cells in tumor nodules (Supplemental Figure 3, D–F). Similarly, ICC, induced using AKT and Notch1 intracellular domain (NICD) (AKT/NICD) (10), also identified KRT19^+^AXIN2^+^ cells as a key source for ICC progression (Supplemental Figure 3, G–I). Through scRNA-Seq data, we detected high expression of SRY-box transcription factor 4 (*Sox4*) and Kruppel-like factor 6 (*Klf6*) in the C2 epithelial cell cluster, with *Sox4* and *Klf6* being associated with the maintenance of cancer stem cell stemness (12, 13). Furthermore, IF staining confirmed that most Tom^+^ cells were positive for SOX4 and KLF6 (Figure 2, H–N). GSVA identified key markers for the other 3 subclusters: ATPase H^+^ transporting V1 subunit F (*Atp6v1f*), sterol carrier protein 2 (*Scp2*), and marker of proliferation Ki-67 (*Mki67*). IF staining showed a gradual increase in Tom^+^ATP6V1F^+^, Tom^+^SCP2^+^, and Tom^+^EdU^+^ cells from day 3 to week 3 after tamoxifen labeling (Figure 2, O–R). Furthermore, limiting dilution assays demonstrated higher tumorigenic potential in Tom^+^ cells compared with Tom ^–^ ICC cells (Figure 2, S–U).

**Figure 3:**
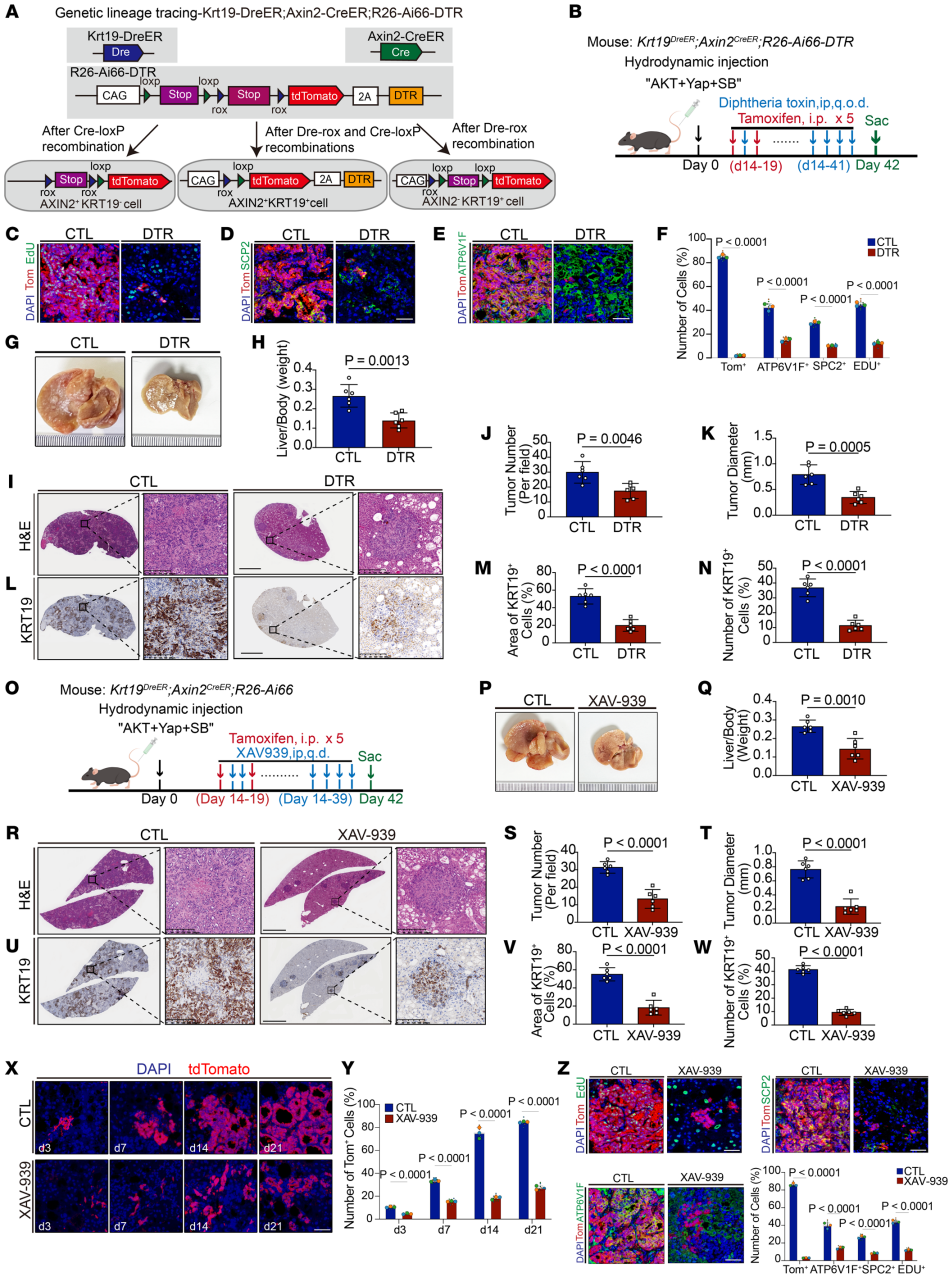
WNT-activated cells and WNT/β-catenin signaling are responsible for murine ICC progression. (**A**) Schematic of DTR-mediated ablation of KRT19^+^AXIN2^+^ cells. (**B**) Experimental strategy for lineage ablation of KRT19^+^AXIN2^+^ cells. Sac, sacrifice. (**C**–**E**) Fluorescence staining for EdU (green, **C**), SCP2+ (green, **D**), ATP6V1F^+^ (green, **E**), and Tom^+^ (red) cells in ICC mice after DT treatment. Scale bars: 50 μm. (**F**) Quantification of Tom^+^, Tom^+^EdU^+^, Tom^+^SCP2^+^, and Tom^+^ATP6V1F^+^ cells in ICC mice after DT treatment. (**G**) Representative images of liver morphology after DT treatment. (**H**) Statistical analysis of liver-to-body weight ratio after DT treatment. (**I**–**N**) Representative images of H&E (**I**) and KRT19 (**L**) staining of liver sections after tamoxifen and DT treatment. Scale bars: 200 μm. Statistical analyses: ICC number (**J**), ICC diameter (**K**), KRT19^+^ cell area (**M**), and KRT19^+^ cell number (**N**). (**O**) Experimental strategy for XAV-939 treatment in ICC mice. (**P**) Representative image of liver morphology after XAV-939 treatment. (**Q**) Statistical analysis of liver to body weight ratio after XAV-939 treatment. (**R**–**W**) Representative images of H&E (**R**) and KRT19 (**U**) staining of liver sections after XAV-939 treatment. Scale bars: 200 μm. Statistical analyses: ICC number (**S**), ICC diameter (**T**), KRT19^+^ cell area (**V**), and KRT19^+^ cell number. (**X** and **Y**) Fluorescence images of lineage tracing at days 3, 7, 14, and 21 in ICC tumors after XAV-939 treatment, with nuclei stained with DAPI (blue). Scale bar: 50 μm (**X**). Quantification of Tom^+^ cells at these time points (**Y**). (**Z**) Fluorescence staining of EdU^+^, SCP2^+^, and ATP6V1F^+^ (green) and Tom^+^ (red) cells in ICC mice after XAV-939 treatment. Scale bars: 50 μm. Quantification of Tom^+^, EdU^+^, SCP2^+^, and ATP6V1F^+^ cells in ICC tumors (bottom right). Data represent the mean ± SD (**F**, **H**, **J**, **K**, **M**, **N**, **Q**, **S**, **T**, **V**, **W**, **Y**, and **Z**). *P* values were calculated by 2-tailed, unpaired Student’s *t* test for **F**, **H**, **J**, **K**, **M**, **N**, **Q**, **S**, **T**, **V**, **W**, **Y**, and **Z**.

To examine whether targeting KRT19^+^AXIN2^+^ cells in ICC impaired ICC progression, we generated *Krt19-DreER Axin2-CreER R26-Ai66-DTR* (DTR hereafter) mice (Figure 3A) (14). In this model, 2 weeks after hydrodynamic injection, tamoxifen and diphtheria toxin (DT) treatment enabled ablation of KRT19^+^AXIN2^+^ cells (Figure 3B). Results showed depletion of KRT19^+^AXIN2^+^ cells and a decrease in the other 3 cell subclusters, as confirmed by IF staining for red fluorescent protein (RFP), EdU, SCP2, and ATP6V1F (Figure 3, C–F). This dramatically inhibited liver indices, measured as liver-to-body weight ratios (Figure 3, G and H). Moreover, ablation of KRT19^+^AXIN2^+^ cells resulted in fewer and smaller ICC tumors than was seen in controls (Figure 3, I–K). Consistently, IHC of KRT19 revealed markedly reduced KRT19^+^ signals in the treatment group (Figure 3, L–N). The number of MKI67^+^ cells markedly decreased, whereas apoptosis (active caspase 3) increased in ICC after KRT19^+^AXIN2^+^ cell ablation compared with controls (Supplemental Figure 3, K–N).

Next, we investigated the role of canonical WNT/β-catenin signaling in maintaining TICs in murine ICC by treating *Krt19-DreER, Axin2-CreER, R26-Ai66*, and WT mice with XAV-939, a potent tankyrase inhibitor targeting WNT/β-catenin signaling (Figure 3O). Western blot analysis showed that XAV-939 suppressed the elevation of phosphorylated LRP6 (p-LRP6), total β-catenin, and nuclear β-catenin, and decreased the levels of p–β-catenin levels in ICC TICs, with no change in LRP6 expression (Supplemental Figure 3J), indicating effective inhibition of WNT/β-catenin signaling. XAV-939 treatment suppressed ICC development (Figure 3, P–W, and Supplemental Figure 3, O–R). Lineage tracing revealed reduced the number of Tom^+^ cells (Figure 3, X and Y), suggesting that WNT/β-catenin signaling was essential for KRT19^+^AXIN2^+^ TICs in ICC. Additionally, IF staining revealed decreased C0, C1, and C3 subclusters upon WNT/β-catenin inhibition (Figure 3Z).

### Cell-cell interactions between ICC epithelial cells and other cell types.

**Figure 4:**
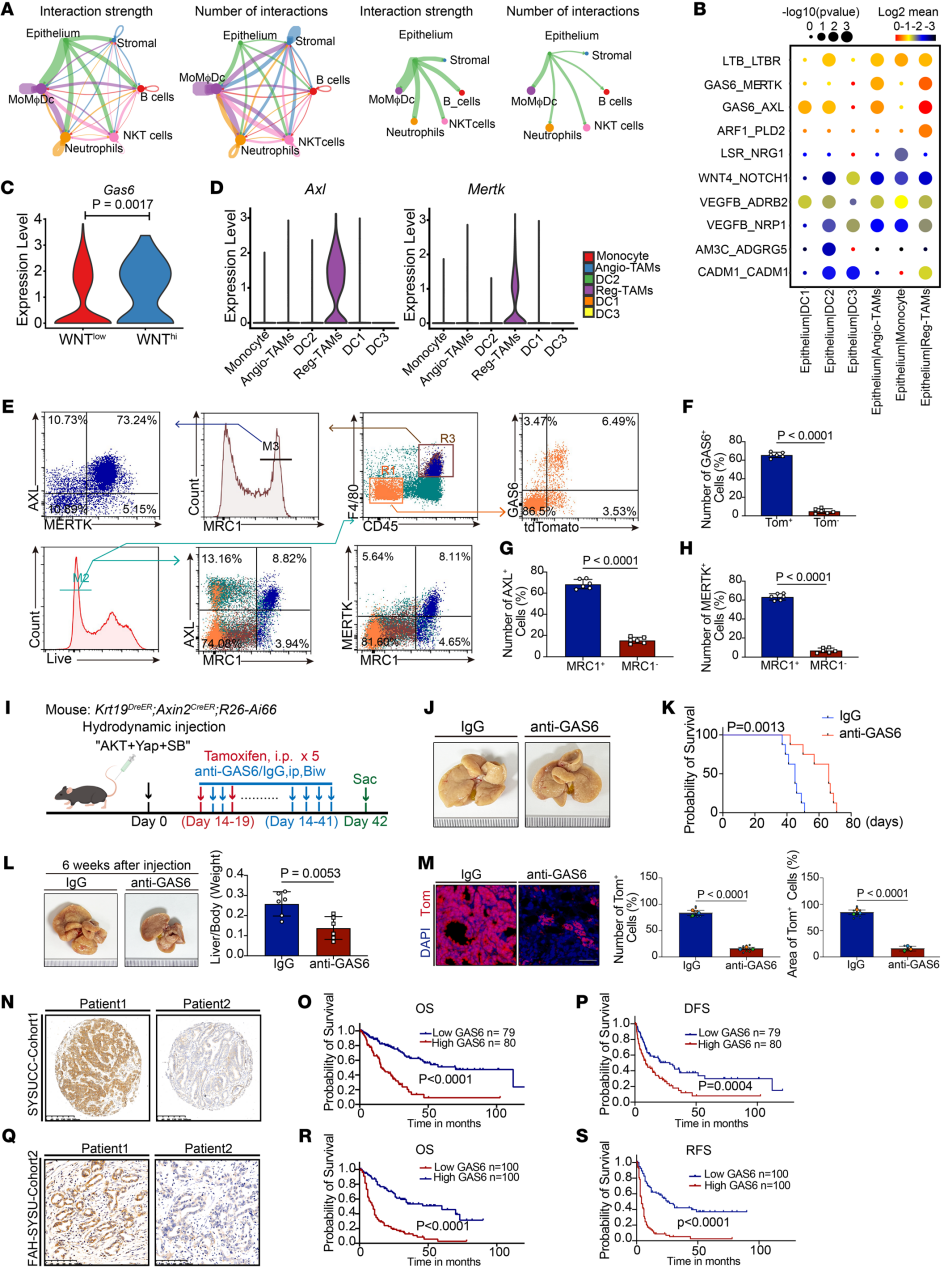
GAS6 is highly expressed and plays an important role in TIC maintenance in ICC. (**A**) Circle plots show interaction strength and number of interactions in cell-cell communication among 6 major clusters (left) using CellChat and between epithelial and other cells (right). Line width correlates with communication probability. (**B**) Dot plots depict the significance (–log_10_ *P* value) and strength (log_2_ mean value) of detailed ligand-receptor pairs between epithelial and other cell types analyzed by CellphoneDB. (**C**) Violin plot displays the *Gas6* expression differences between WNT^hi^ and WNT^lo^ epithelial cell subclusters. (**D**) Violin plots show *Axl* and *Mertk* expression in MoMϕDC subclusters. (**E**–**H**) Flow plots (**E**) show Tom^+^GAS6^+^ tumor-infiltrating cells and the levels of MRC1, MERTK, and AXL expression in infiltrating Reg-TAMs. Graphs depict statistical analysis of GAS6^+^ in Tom^+/–^ cells in mouse ICC (**F**) as well as AXL^+^ (**G**) and MERTK^+^ (**H**) expression in MRC1^+/–^ cells in ICC. (**I**) Treatment strategy with anti-GAS6/IgG in ICC mice. (**J**) Representative liver morphology image for survival outcome analysis after anti-GAS6 treatment. (**K**) Kaplan-Meier OS curve for ICC mice after anti-GAS6 treatment. (**L**) Representative liver morphology image after 6 weeks of anti-GAS6 treatment (left) and statistical analysis of liver-to-body weight ratio (right). (**M**) Fluorescence images of lineage tracing (left) (scale bar: 50 μm). Statistical analysis of Tom^+^ cell numbers (middle), and Tom^+^ cell area (right) after treatment. (**N**–**S**) Representative images of GAS6 staining in human ICC tissue from SYSUCC cohort 1 (**N**) (scale bar: 200 μm) and FAHSYSU cohort 2 (**Q**) (scale bar: 100 μm). Kaplan-Meier curves based on GAS6 expression in ICC: OS and DFS for SYSUCC cohort 1 (**O** and **P**), and OS and RFS for FAHSYSU cohort 2 (**R** and **S**). Data represent the mean ± SD (**F**–**H**, **L**, and **M**). *P* value were calculated by 2-tailed,, unpaired Student’s *t* test (**C**, **F**–**H**, **L**, and **M**), log-rank test (**K**, **O**, **P**, **R**, and **S**).

Emerging evidence shows that the TME regulates the plasticity and immune escape of TICs (15). To investigate cell interactions in ICC, we constructed a cell-cell communication network using CellChat and identified distinct interactions (Figure 4A). Notably, mouse ICC epithelial cells exhibited stronger ligand-receptor interactions with MoMϕDC populations (Figure 4A). To investigate these interactions in detail, mouse MoMϕDCs were partitioned into subclusters: monocytes, Reg-TAMs, angiogenic activity TAMs (Angio-TAMs), DC1, DC2, and DC3 (Supplemental Figure 4A), similar lineages were found in human MoMϕDCs (Supplemental Figure 4C). Gene expression for major myeloid subsets is shown in Supplemental Figure 5, B and D. Unsupervised clustering of mouse and human myeloid subclusters using orthologous genes revealed a 1-to-1 relationship (Supplemental Figure 4, E and F).

Then, we conducted CellphoneDB analysis to map ligand-receptor pairs and found that GAS6-AXL-MERTK interactions, which promote the immunosuppressive TME (16), were highly enriched between ICC epithelium and Reg-TAMs (Figure 4B). Furthermore, we found that *Gas6* expression was markedly elevated in the WNT^hi^ epithelial subcluster compared with the WNT^lo^ epithelial subclusters (Figure 4C). In both mouse and human samples, *Axl*/*Mertk* (*AXL*/*MERTK*) were predominantly expressed in Reg-TAMs (Figure 4D and Supplemental Figure 4G). Flow cytometric analysis of ICC samples 3 days after lineage tracing showed that GAS6 levels were higher in Tom^+^ ICC cells than in Tom^–^ ICC cells (Figure 4, E and F), and AXL and MERTK were enriched in Reg-TAMs (Figure 4, G and H). Additionally, human ICC scRNA-Seq data showed higher CytoTRACE scores and upregulated *GAS6* (Supplemental Figure 2C). Both human ICC scRNA-Seq and TCGA-CHOL data revealed higher *GAS6* expression in tumor cells, with positive correlations to WNT signaling genes (Supplemental Figure 4, H–J).

### GAS6-AXL-MERTK interactions between epithelial cells and Reg-TAMs play important roles in mouse ICC development.

To explore the role of GAS6-AXL-MERTK interactions in ICC, we treated *Krt19-DreER Axin2-CreER R26-Ai66* ICC mice and WT ICC mice with a GAS6 neutralizing antibody (Figure 4I). GAS6 antibody–treated mice exhibited delayed tumor burden and improved survival (median of 66 days) compared with controls (median of 45 days) (Figure 4, J and K). After 6 weeks, GAS6 antibody treatment markedly reduced the number of Tom^+^ cells (Figure 4, L and M) and decreased the number of KRT19^+^ and MKI67^+^ cells (Supplemental Figure 5, A–H). Histological analysis also revealed an increase in active caspase 3^+^ cells, suggesting the key role of GAS6 in cholangiocarcinogenesis (Supplemental Figure 5, I and J).

To explore the prognostic value of GAS6 in ICC, we performed IHC staining of patient ICC tissue microarrays from 2 cohorts (see clinical characteristics in Supplemental Tables 1 and 2). Tumors were categorized into high and low GAS6 groups using the median GAS6^+^ signal as the cutoff. Representative images of GAS6 IHC staining for high- and low-density positive signals are presented in Figure 4, N and Q. In the Sun Yat-Sen University Cancer Center (SYSUCC) cohort (*n* = 159), the GAS6^hi^ group had markedly poorer overall survival (OS) and disease-free survival (DFS) than did the GAS6^lo^ group (Figure 4, N–P). Similar results were observed in the FAHSYSU cohort (*n* = 200) (Figure 4, Q–S). High GAS6 levels were also substantially associated with tumor stage and grade (Supplemental Figure 5, K and L). We generated *Axin2-creER Gas6^fl/fl^* mice and conducted AKT/YAP/SB hydrodynamic injection (Supplemental Figure 6A). KO of *Gas6* was validated by Western blotting (Supplemental Figure 6I). Conditional deletion of *Gas6* in Axin2^+^ cells improved ICC phenotype severity (Supplemental Figure 6, B–H). Flow cytometry revealed reduced CD4^+^ T cell and Treg infiltration, with increased CD8^+^ T cells in ICC tumors from *Axin2-creER Gas6^fl/fl^* mice, while NK and NKT cell frequencies remained unchanged (Supplemental Figure 6, J–Q). To investigate TIC-derived GAS6 suppression of tumor immunity via AXL/MERTK on Reg-TAMs, we used *Krt19-DreER Axin2-creER R26-Ai66* mice for AKT/YAP/SB hydrodynamic injection and sorted Reg-TAMs and epithelial cell adhesion molecule^+^/Tom^+^ (EpCAM^+^Tom^+^) TICs. Coculturing results showed elevated AXL, MERTK, and AKT phosphorylation in Reg-TAMs with TICs (Supplemental Figure 6, R and S), confirming that GAS6 on TICs promoted AXL, MERTK, and AKT phosphorylation in Reg-TAMs (17).

**Figure 5:**
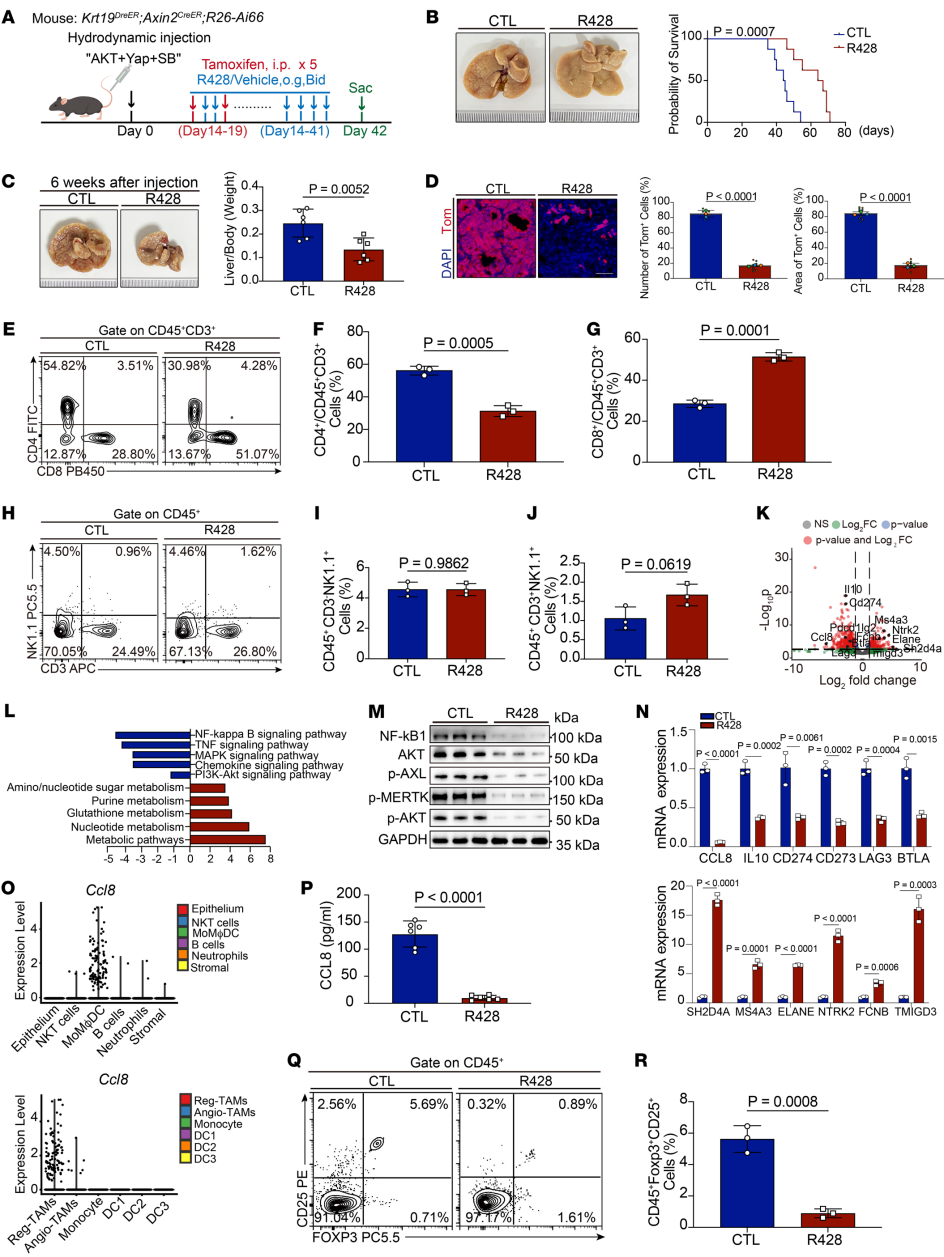
Inhibition of AXL/MERTK suppresses ICC progression and Treg numbers. (**A**) Experimental strategies for R428/Vehicle treatment in ICC mice. (**B**) Representative liver morphology image of different treatment groups for survival outcome analysis (left). Kaplan-Meier survival curve for ICC mice after R428 treatment (right). (**C**) Representative image of liver morphology after R428 treatment for 6 weeks (left). Statistical analysis of liver to body weight ratio after R428 treatment (right). (**D)** Fluorescence images of lineage tracing after R428 treatment (left). Scale bar: 50 μm. Statistical analysis of Tom^+^ cell number (middle) and area (right) after treatment. (**E**–**G**) Flow plots (**E**) and graphs of tumor-infiltrating CD4^+^ (**F**) and CD8^+^ (**G**) T cell frequency after R428 treatment. (**H**–**J**) Flow plots (**H**) and graphs of tumor-infiltrating NK (**I**) and NKT (**J**) cell frequency after R428 treatment. (**K**) Volcano plot of differentially expressed genes between R428- and vehicle-treated murine ICC Reg-TAMs, with genes meeting *P* < 0.01 and fold change ≥2 or ≤–2 shown in red. (**L**) KEGG pathway analysis of the up- and downregulated differentially expressed genes in ICC tumor cells after R428 treatment. (**M**) Western blot analysis of NF-κB1, AKT, p-AXL, p-MERTK, p-AKT, and GAPDH in ICC Reg-TAMs after R428 treatment. (**N**) qRT-PCR analysis of differentially expressed genes in ICC tumor cells after R428 treatment. (**O**) Dot plots showing specific expression of Ccl8 in MoMϕDC cluster (top) and Reg-TAMs (bottom). (**P**) ELISA showed CCL8 protein levels in ICC Reg-TAMs between R428 and vehicle treatment groups. (**Q** and **R**) Representative flow plots (**Q**) and Treg frequency graph (**R**) after R428 treatment. Data represent the mean ± SD (**C**, **D**, **F**, **G**, **I**, **J**, **N**, **P**, and **R**). *P* values were calculated by 2-tailed, unpaired Student’s *t* test (**C**, **D**, **F**, **G**, **I**, **J**, **N**, **P**, and **R**) and log-rank test (**B**). CTL, control.

To explore the role of AXL/MERTK in Reg-TAMs and ICC development, we treated ICC-bearing mice with R428, an inhibitor of tyrosine kinases, including AXL and MERTK (17). R428-treated mice showed substantially reduced tumor burden and improved survival (Figure 5, A–C, and Supplemental Figure 7, A–F). Cell proliferation was moderately inhibited after 6 weeks of treatment (Supplemental Figure 7, G and H), whereas apoptosis markedly increased (Supplemental Figure 7, I and J). To assess the effect of R428 inhibition on the interaction between Reg-TAMs and TICs, EpCAM^+^Tom^+^ TICs and Reg-TAMs from ICC-bearing *Krt19-DreER* *Axin2-creER* *R26-Ai66* mice were sorted and cocultured with or without R428. Western blotting showed downregulation of p-AXL, AKT, p-AKT, p-MERTK, and NF-κB1 in Reg-TAMs (Supplemental Figure 7K). Lineage-tracing assays revealed reduced expansion of TICs in R428-treated mice (Figure 5D). In addition, *Lyz2-creER Axl^fl/fl^ Mertk^fl/fl^* mice (*Axl* and *Mertk* conditional double-KO [cDKO]) mice were generated and used for AKT/YAP/SB hydrodynamic injection (Supplemental Figure 7P). The efficiency of the DKO was confirmed by Western blotting (Supplemental Figure 8A). As expected, KO of *Axl* and *Mertk* in myeloid cells alleviated ICC phenotype severity (Supplemental Figure 7, Q–W).

Since AXL/MERTK are mainly expressed in mouse Reg-TAMs, we conducted flow cytometry to examine tumor-infiltrated macrophages. Flow cytometry revealed that the number of CD11b^+^ F4/80^+^ macrophages in R428-treated ICC samples was comparable to that of controls (Supplemental Figure 7, L and M, and Supplemental Figure 14A). Surprisingly, the percentages of F4/80^+^MRC1^+^ and F4/80^+^CD86^+^ double-positive cells were similar (Supplemental Figure 7, N and O), indicating that AXL/MERTK inhibition did not affect macrophage polarization. This was further confirmed in ICC samples treated with a GAS6 antibody (Supplemental Figure 5, M–P). Moreover, *Axl* and *Mertk* DKO in ICC did not alter macrophage numbers or polarization (Supplemental Figure 8, C–F). Our data suggest that AXL/MERTK signaling may not be essential for the number and polarization of macrophages.

Given that the R428 treatment inhibited mouse ICC tumor growth, we examined the major tumor-infiltrating T lymphocytes. Tumors from R428-treated mice showed a reduction in CD4^+^ T cells and an increase in CD8^+^ T cells (Figure 5, E–G, and Supplemental Figure 14B). However, no marked differences were observed in CD45^+^CD3^+^NK1.1^+^ NKT cells or CD45^+^CD3^–^NK1.1^+^ NK cells (Figure 5, H–J). Similarly, *Axl* and *Mertk* DKO reduced the proportion of CD4^+^ T cells in ICC samples without marked changes in NK/NKT cell numbers (Supplemental Figure 8, G–L). To dissect the functional link between AXL/MERTK in Reg-TAMs and T cells in ICC, we isolated Reg-TAMs from R428-treated ICC for RNA-Seq. We identified 483 upregulated and 1,001 downregulated genes in R428-treated Reg-TAMs (Figure 5K and Supplemental Table 3). KEGG analysis revealed that downregulated genes were primarily involved in the NF-κB, TNF, MAPK, chemokine, and PI3K/AKT pathways (Figure 5L). Western blotting confirmed reduced levels of p-AXL, p-AKT, AKT, p-MERTK, and NF-κB1 in Reg-TAMs after R428 treatment (Figure 5M) and quantitative reverse transcription PCR (qRT-PCR) verified the repression of target genes (Figure 5N).

To identify the potential functional mediator of AXL/MERTK in Reg-TAMs, we examined the top 10 downregulated genes in both mouse and human scRNA-Seq datasets. We found that *Ccl8* was the only secreted factor specifically expressed in Reg-TAMs (Supplemental Table 3, Figure 5O, and Supplemental Figure 9A). R428 treatment substantially reduced CCL8 levels in Reg-TAMs, as confirmed by ELISA (Figure 5P). Similarly, both ELISA and Western blotting showed reduced CCL8 levels after *Axl* and *Mertk* DKO (Supplemental Figure 8, A and C). *CCL8* expression strongly correlated with *CD163*, *AXL*, and *MERTK* in the TCGA-CHOL dataset (Supplemental Figure 9B). It has been shown that CCL8 affects Treg infiltration (18), and we observed a marked decrease in Tregs in the R428-treated group (Figure 5, Q and R, and Supplemental Figure 9C). Similarly, *Axl* and *Mertk* DKO also reduced Treg numbers (Supplemental Figure 8, N and Q).

### CCL8 is one of the key mechanisms of the GAS6/AXL/MERTK signaling pathway in ICC formation.

**Figure 6:**
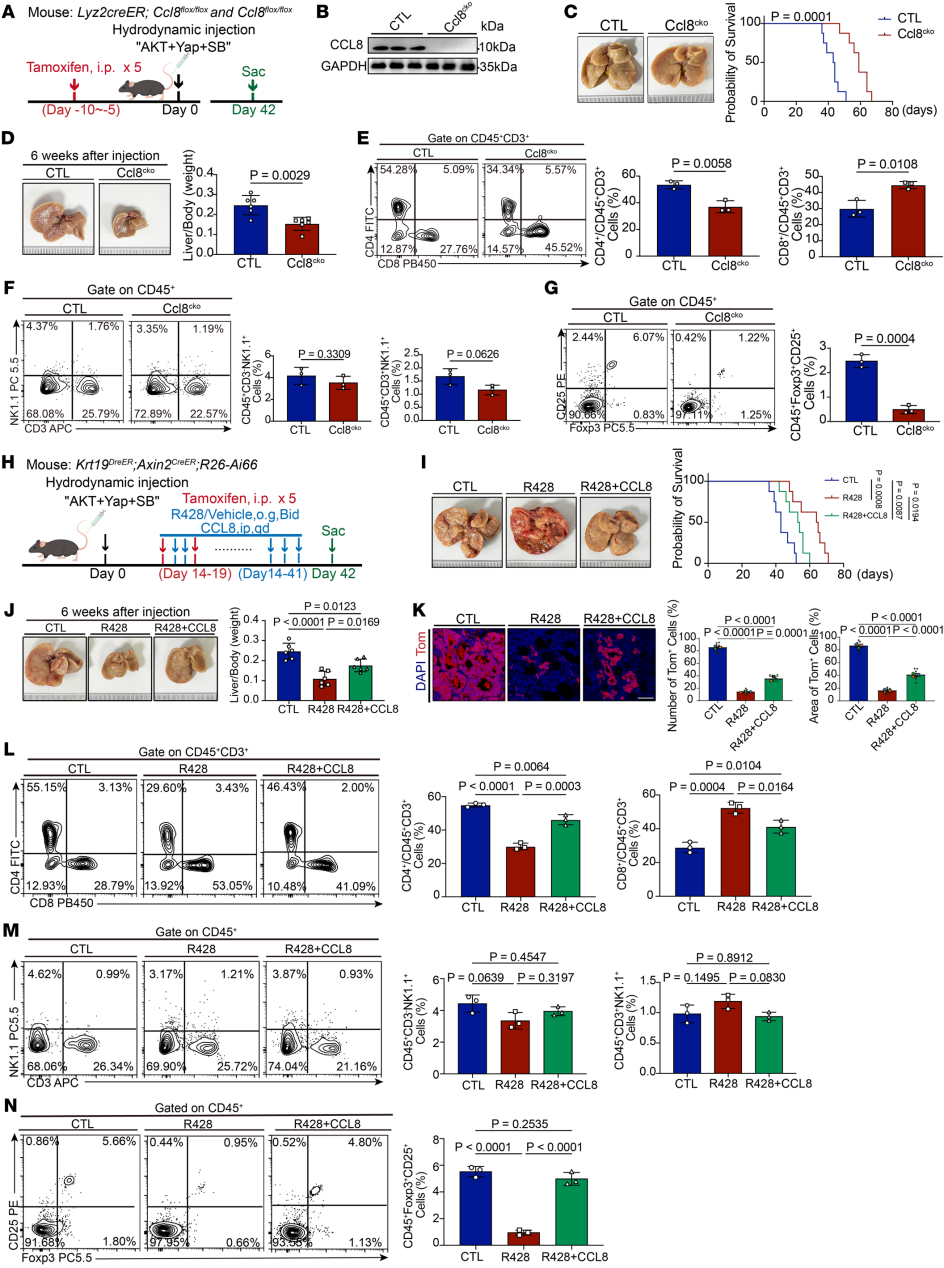
CCL8 is the downstream mediator of AXL/MERTK signaling in Reg-TAMs. (**A**) Experimental design for induction of ICC in Lyz2-CreER (Control, CTL) and Lyz2-CreER *Ccl8^fl/fl^* (Ccl8cko) mice. (**B**) The KO efficiency of CCL8 in Lyz2+cells was validated by Western blotting. (**C**) Representative liver morphology images of control and Ccl8cko mice for survival outcome analysis (left). Kaplan-Meier OS curve for control and Ccl8cko mice is shown (right). (**D**) Representative liver morphology images of control and Ccl8cko mice after 6 weeks of plasmid injection (left) and statistical analysis of liver-to-body weight ratio (right). (**E**–**G**) Flow plots show immune cell frequencies and counts in control and Ccl8cko mice: CD4^+^ and CD8^+^ T cells (**E**), NK and NKT cells (**F**), and Tregs (**G**). Each plot displays cell frequencies (left) and quantitative analysis (right). (H) Experimental strategy for ICC mice using R428 alone or R428 with CCL8 (R428+CCL8). (**I**) Representative liver morphology image of different treatment groups for survival outcome analysis (left). Kaplan-Meier OS curve for mice with ICC in different treatment groups (right). (**J**) Liver morphology (left) in different treatment groups after 6 weeks of treatment and statistical analysis of liver-to-body weight ratios (right). (**K**) Fluorescence images (left) of lineage tracing in different treatment groups, statistical analysis of Tom^+^ cell number and areas (right); Scale bar: 50 μm. (**L**–**N**) Flow plots (left) and graphs (right) showing frequencies of tumor-infiltrating CD4^+^ and CD8^+^ T cells (**L**), NK and NKT cells (**M**), and Tregs (**N**) in different treatment groups. Data represent the mean ± SD (**D**–**G** and **J**–**N**). *P* values were calculated by 2-tailed, unpaired Student’s *t* test (**D**–**G**), 1-way ANOVA with Tukey’s multiple-comparison test (**J**–**N**), and log-rank test (**C** and **I**).

Then, to assess the effects of CCL8 on mouse ICC, we generated *Lyz2-creER Ccl8^fl/fl^* mice for AKT/YAP hydrodynamic injection (Figure 6A). As expected, *Ccl8* deletion in myeloid cells prolonged survival and reduced the severity of ICC (Figure 6, B and C, and Supplemental Figure 10, A–F). The frequency of NK and NKT cells remained unchanged, whereas CD4^+^ T cells and Tregs decreased and CD8^+^ T cells increased (Figure 6, D–G). Additionally, flow cytometry showed a slight reduction in the infiltration of conventional DCs (cDCs), plasmacytoid DCs (pDCs), monocytes, macrophages, and polymorphonuclear (PMN) cells in ICC tumor samples from *Lyz2-creER* *Ccl8^fl/fl^* mice (Supplemental Figure 10, G–P). Moreover, we used *Ccl8^–/–^* mice for AKT/YAP hydrodynamic injection (Supplemental Figure 9C). The efficiency of *Ccl8* KO was confirmed by Western blotting (Supplemental Figure 9D). Notably, *Ccl8* deletion led to prolonged survival and reduced ICC severity (Supplemental Figure 9, E–P). Consistently, flow cytometric analysis showed a decrease in CD4^+^ T cells and Tregs, an increase in CD8^+^ T cells, and unchanged NK and NKT cells in ICC samples from *Ccl8^–/–^* mice (Supplemental Figure 9, Q–X).

We then wondered whether CCL8 overexpression could reverse the effects of R428 on mouse ICC. Mice treated with R428 and systemic CCL8 overexpression exhibited notably reversed delayed ICC formation and prolonged survival induced by R428 treatment (Figure 6, H–J). Lineage-tracing assays revealed that CCL8 reversed the inhibition of KRT19^+^AXIN2^+^ TIC expansion in R428-treated mice (Figure 6K). Flow cytometry showed that CCL8 substantially affected the infiltration of Tregs, CD4^+^ T cells, and CD8^+^ T cells but not of NK or NKT cells (Figure 6, L–N). Consistently, histological and IHC staining confirmed that CCL8 rescued the inhibition of tumor formation and apoptosis induction by R428, with cell proliferation largely unchanged (Supplemental Figure 12, K–N). Similarly, in *Lyz2-creER* *Axl^fl/fl^* *Mertk^fl/fl^* mice, *Axl* and *Mertk* DKO led to a slight reduction in the infiltration of cDCs, pDCs, monocytes, and PMN cells, but these changes showed no marked differences. Furthermore, CCL8 administration after the DKO did not notably affect the infiltration of these cells (Supplemental Figure 11). These results suggest that AXL/MERTK signaling promoted ICC formation partially via CCL8. To evaluate whether *Axl* and *Mertk* in macrophages influence the function of CD8^+^ T cells and Tregs, further coculturing of Reg-TAMs with CD8^+^ T and naive CD4^+^ T cells showed no notable differences in CD8^+^ T cell proliferation or Treg differentiation, regardless of *Axl* and *Mertk* DKO or CCL8 addition (Supplemental Figure 12, A and B).

### Inhibition of GAS6/AXL/MERTK signaling sensitizes murine ICC cells to anti–PD-1 treatment.

**Figure 7:**
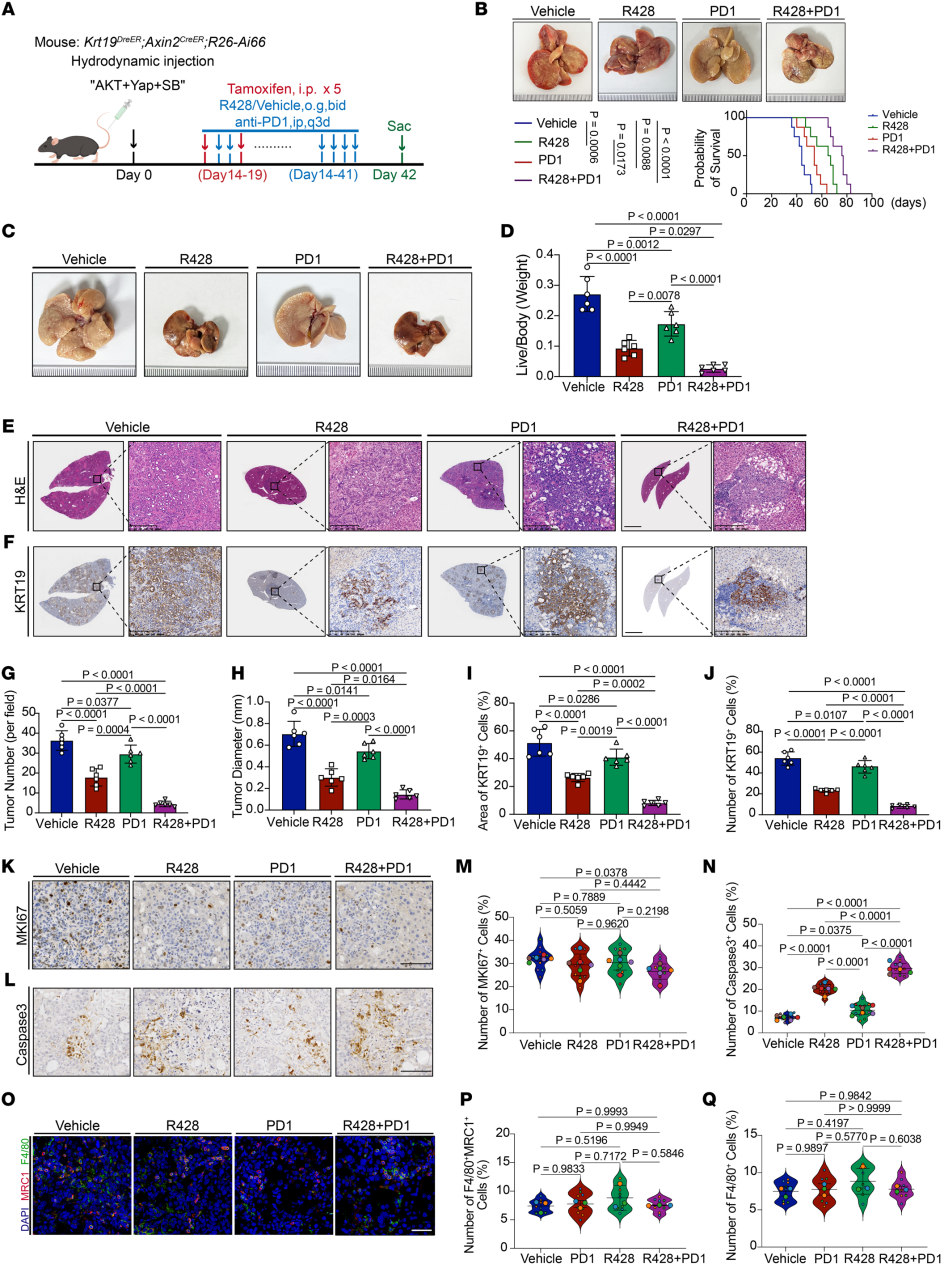
R428 treatment sensitizes murine ICC cells to anti–PD-1 treatment. (**A**) Experimental strategies for ICC mice with the indicated treatment. The mice were sacrificed when large tumors developed. (**B**) Representative liver morphology images from ICC mice under the indicated treatment for survival outcome analysis (left). The time point at which mice developed lethal tumor burden is shown. Kaplan-Meier OS curve for mice with ICC subjected to the indicated treatment. *P* values were calculated by log-rank test. (**C** and **D**) Representative images of whole liver morphology from ICC mice subjected to the indicated treatment. Statistical analysis of liver-to-body weight ratio from ICC mice with the indicated treatment. Values represent the mean ± SD from 6 independent biological replicates (*n* = 6 mice). *P* values were calculated by 1-way ANOVA with Tukey’s multiple-comparison test. (**E**–**J**) Representative images of H&E (**E**) and KRT19 (**F**) staining of liver sections from ICC mice under different treatments. Scale bars: 200 μm. Statistical analyses of ICC tumor number (**G**), ICC tumor diameter (**H**), KRT19^+^ area (**I**), and KRT19^+^ cells (**J**) in different treatment groups. Values represent the mean ± SD from 6 independent biological replicates (*n* = 6 mice). *P* values were calculated by 1-way ANOVA with Tukey’s multiple-comparison test. (**K**–**N**) Representative images of MKI67 (**K**) and active caspase 3 (**L**) staining of liver sections from ICC mice under the indicated treatments. Scale bars:100 μm. Statistical analyses of MKI67^+^ cells (**M**) and active caspase 3^+^ cells (**N**). Values represent the mean ± SD from 6 independent biological replicates (*n* = 12 fields from 6 mice). *P* values were calculated by 1-way ANOVA with Tukey’s multiple-comparison test. (**O**–**Q**) Fluorescence staining for F4/80 (green) and MRC1 (red) expression in liver sections from ICC mice under the indicated treatments. Scale bar: 50 μm (**O**). (**P** and **Q**) Statistical analyses of F4/80^+^MRC1^+^ cells and F4/80^+^ cells. Data represent the represent the mean ± SD from 3 independent experiments (*n* = 9 fields from 3 mice). *P* values were calculated by 1-way ANOVA with Tukey’s multiple-comparison test.

The markedly decreased Treg infiltration in murine ICC tumors after R428 treatment encouraged us to investigate whether combining R428 with anti–programmed cell death 1 (anti–PD-1) therapy could further inhibit tumor growth. ICC-bearing mice were treated with vehicle, R428, anti–PD-1, or R428 plus anti–PD-1 (Figure 7A). All treatments suppressed tumor growth compared with vehicle treatment, but anti–PD-1 monotherapy only modestly improved survival and reduced tumor burden. In contrast, R428 monotherapy and combination therapy had more pronounced effects (Figure 7, B–J). Analysis of tumor tissues revealed that MKI67^+^ cell percentages were reduced only in the combination group, while active caspase 3^+^ cell percentages were highest in the combination group (Figure 7, K–N). TAM frequency and Reg-TAM percentages were unchanged (Figure 7, O–Q). Flow cytometry showed that R428 or R428 plus anti–PD-1 reduced Treg infiltration and increased CD8^+^ T cell infiltration, while anti–PD-1 alone did not notably affect Tregs and only slightly increased CD8^+^ T cell percentages (Supplemental Figure 13).

## Discussion

TICs contribute to intratumoral heterogeneity and high mortality in human and mouse ICC. We reported immune-evasive, WNT-responsive TICs in ICC and their crosstalk with Reg-TAMs via the GAS6/AXL/MERTK pathway. This interaction enables TICs to evade destruction by adaptive immunity through Treg recruitment via CCL8 cytokine secretion. Our study reveals a mechanism by which TICs in ICC establish immune evasion.

In this study, we performed scRNA-Seq to investigate the molecular mechanism of TICs in ICC using an AKT/YAP hydrodynamic injection mouse model, which recapitulates the morphological and molecular features of human ICC (9, 10). A comparison of our mouse and human ICC scRNA-Seq datasets revealed substantial overlap in cellular composition and molecular signatures. Trajectory inference consistently showed WNT-activated malignant cells at the top of the ICC progression hierarchy, suggesting they may be potential TICs. Few studies have used lineage tracing to explore ICC TICs. Combining a dual-recombinase lineage-tracing system and a cellular ablation assay, we found substantial evidence to support the idea that WNT-activated cells are the bone fide TICs in ICC.

Previous reports show that, while non-TICs can form tumors in highly immunocompromised mice but fail to do so in partially immunocompromised mice, TICs can give rise to tumors in both scenarios, suggesting that immune evasion is one of the key features that distinguish TICs from non-TICs (5). The interaction between TIC stemness and immunogenicity in ICC remains largely unexplored. TICs escape immunosurveillance through mechanisms such as upregulation of immune checkpoints, repression of T cell activation, and downregulation of MHC class I expression (19, 20). TAMs play a critical role in TIC immune evasion through reciprocal interactions. TICs recruit and activate macrophage precursors via chemokines and periostin (21, 22), whereas TAMs support TIC maintenance by secreting cytokines like IL-6, IL-1β, TNF-α, TGF-β, CCL2, and CCL5 (23). Macrophages are traditionally divided into M1 and M2 types. M1 macrophages (CD86^+^CD80^+^) are critical for suppressing tumor growth, while M2 macrophages (CD206^+^CD163^+^) display protumor properties (24). However, in our analysis and online scRNA-Seq datasets, we found it challenging to distinguish M1-M2 polarization in ICC TAMs. Instead, we identified Reg-TAMs expressing *Mrc1*, *C1qa*, *C1qb*, and *C1qc*. Remaining Angio-TAMs expressed *Arg1*, *Bnip3*, and *Mif*, showing an intermediate phenotype between monocytes and Reg-TAMs.

In our study, we showed that treatment with GAS6 neutralizing antibody led to inhibited expansion of WNT-activated TICs in mouse ICC. Previously, GAS6 has been shown to be a potent factor for β-catenin stabilization and subsequent T cell factor/lymphoid enhancer–binding factor (TCF/LEF) transcriptional activation (25). GAS6 seemed to determine WNT activity in TICs in ICC. However, the upstream event of GAS6 remains unknown. It will be helpful to determine whether altering the genomic landscape relevant to GAS6 might be a driving event of ICC initiation in a larger patient cohort. In addition, the extent to which GAS6 inhibition can directly inhibit the function of TICs requires further investigation, given that blocking GAS6 also caused marked changes in the TME that were heavily involved in TIC regulation. Notably, GAS6 expression was highly correlated with WNT-related genes in the TCGA-CHOL datasets. Additionally, GAS6 may be a prognostic marker for ICC, based on results from 2 independent cohorts.

The PD-1/programmed death ligand 1 (PD-1/PD-L1) pathway and Tregs are critical for TICs to evade immune surveillance and suppress antitumor immunity (26, 27). We found that treatment with R428 synergized with anti–PD-1 therapy in murine ICC, resulting in the infiltration of fewer numbers of Tregs, partially due to inhibition of CCL8. The exact details of how R428 modulates Treg infiltration have not been fully explored. A previous study has shown that CCL8 recruits CCR5^+^ Tregs (18). In addition, ablation of tumor-infiltrating Tregs expressing CCR8, one of the primary receptors for CCL8, can elicit antitumor immunity and improve the efficacy of anti–PD-1 therapy (28). In the future, functional assays will be helpful to validate the role of these receptors in Tregs.

In conclusion, our study highlights the critical role of GAS6-AXL-MERTK interactions between TICs and TAMs in mediating TIC immune evasion in ICC. These findings provide insights into how TICs manipulate TAMs to hijack the immune system. Blocking AXL/MERTK with R428 substantially reduced Treg recruitment to tumors. Consequently, R428 combined with anti–PD-1 therapy dramatically suppressed ICC growth in mice, suggesting that this strategy has promising clinical potential.

## Methods

### Sex as a biological variable.

Both male and female animals were examined in this study, and similar findings were reported for both sexes.

### Constructs and reagents.

The constructs used for mouse hydrodynamic injection in this study, including AKT (pT3-EF1a-HA-myr-AKT, mouse, Addgene 31789), YAP (pT3-EF1a-YAPS127A, human, Addgene 86497), NICD (pT3-EF1a-NICD1, mouse, Addgene 86500), and SB (pCMV-Sleeping Beauty transposase) were courtesy of Xin Chen (UCSF, San Francisco, California, USA). Plasmids were isolated and purified using the Endofree Maxi Plasmid Kit (DP117-TA, Tiangen Biotech).

### Mice, hydrodynamic injection, lineage tracing, and drug treatment.

*Axin2-CreER* (strain no. 018867) mice were purchased from The Jackson Laboratory. C57BL/6, *Krt19-DreER* (strain T056046), *Ccl8^fl/fl^* (strain T013044), *Gas6^fl/fl^* (strain T010051), *Axl^fl/fl^* (strain T009234), *Mertk^fl/fl^* (strain T007888), and *Lyz2-CreER* (strain T052789) mice were purchased from GemPharmatech. The *R26-Ai66-DTR* (NM-KI-190086) mouse strain was purchased from Shanghai Model Organisms. *Ccl8^–/–^* mice were provided by Hongli Zhou (Second Affiliated Hospital of Army Medical University, Chongqing, China). BALB/c-nu/nu mice were purchased from the Laboratory Animal Center at Sun Yat-Sen University. *R26-Ai66-tdTomato* mice were generated as previously described (29). All animals in this study were maintained under specific pathogen–free conditions, housed under a 12-hour light/12-hour dark cycle, and given ad libitum access to food and water. For ICC induction, we hydrodynamically injected 30 μg YAP or 20 μg NICD, along with 4 μg AKT and 1 μg transposase plasmids, into 6- to 10-week-old WT or transgenic mice as previously described (10, 30).

For lineage tracing, tamoxifen (T5648, MilliporeSigma) was dissolved in ethanol and diluted with corn oil to a 10% ethanol/tamoxifen/corn oil mixture at 20 mg/mL. Dre or Cre was activated by i.p. injection of tamoxifen (100 μg/g body weight) for 5 consecutive days. For ablation of DTR-expressing cells, mice were injected i.p. with 500 ng boluses of un-nicked DT (150, List Biologics) every other day. For treatment, XAV-939 (T1878-100, TargetMol) was given i.p. at 2.4 mg/mL in 250 μL once daily (31). R428 (S2841, Selleck Chemicals) was administered by oral gavage at 25 mg/kg twice daily (17). GAS6 neutralizing antibody (AB885, R&D Systems) was injected i.p. at 2 mg/kg twice a week (32). Recombinant MCP-2/CCL8 (HY-P7239, MedChemExpress) was injected at 1 μg in 200 μL PBS once daily (33). Anti–PD-1 antibody (BE0146, Bio X Cell) was administered i.p. at 200 μg/mouse every 3 days (34). Control mice received vehicle or IgG isotype a control antibody. All treatments began the second week after hydrodynamic injection and continued until the day before sample collection.

### Tissue preparation, cell isolation, and scRNA-Seq.

WT mice were euthanized by CO_2_ inhalation 6 weeks after hydrodynamic injection with AKT/YAP/SB for liver collection. Malignant lesions were dissected using a microscope, minced into 1 mm³ pieces, and digested with a Tumor Dissociation Kit (130-096-730, MACS Miltenyi Biotec) at 37°C for 45 minutes using gentleMACS Tissue Dissociator (130-093-235, MACS Miltenyi Biotec). The reaction was stopped with PBS, filtered through a 40 μm Falcon strainer (352340, Corning), and centrifuged at 300*g* for 5 minutes. The pellet was treated with ACK Lysing Buffer (A1049201, Thermo Fisher Scientific) on ice for 5 minutes, and dead cells were removed using MS columns from the Dead Cell Removal Kit (130-090-101, MACS, Miltenyi Biotec). Live cells were resuspended in PBS with 0.04% BSA and counted using the Countess 3 Automated Cell Counter (Thermo Fisher Scientific). Single cells were loaded into a 10× Genomics Chromium Controller (aiming for 20,000 cells per sample), and scRNA-Seq libraries were prepared with Chromium Single Cell 3′ Reagent Version 2 Kit (10× Genomics) and Sequenced on a MGISEQ2000 System (BGI).

### Processing and clustering of scRNA-Seq data.

Following sequencing, raw reads were demultiplexed using the mkfastq command from Cell Ranger (version 4.0.0, 10× Genomics) and aligned to the mm10 reference genome (GRCm38.91) using the count command to generate cell-gene-barcode matrices. These matrices were then merged using Seurat (version 4.0.0) for downstream analysis. Low-quality cells and genes were filtered by excluding genes detected in fewer than 3 cells, cells with fewer than 200 or more than 5,000 genes, less than 10% mitochondrial/hemoglobin gene expression, and doublets identified by DoubletFinder. After quality control, data were integrated and normalized using Seurat’s “harmony” function, retaining 18,410 cells for further analysis.

After data integration and normalization, principal component analysis (PCA) was performed on highly variable genes using the RunPCA function in Seurat. Cell clustering was done with the FindClusters, and the clusters were visualized using uniform manifold approximation and projection (UMAP) with the RunUMAP function. Each cluster was annotated using canonical marker genes identified by the “FindAllMarkers” function with a likelihood ratio test. To assess the cell-cycle status, we applied the “CellCycleScoring” function was applied on the basis of markers for the S and G_2_/M phases.

Available scRNA-Seq datasets were also downloaded from the Genome Sequence Archive (GSA) at the National Genomics Data Center (HRA000863), including 14 pairs of human intrahepatic cholangiocarcinoma (iCCA) tumor and nontumor liver tissues. All quality control, normalization, and downstream analyses were performed using Seurat unless otherwise specified.

Gene correlation analysis within the TCGA-CHOL cohort was performed using Encyclopedia of RNA Interactomes (ENCORI), a state-of-the-art, openly licensed platform for RNA interactome data.

### Bulk RNA-Seq analysis.

Data were filtered using SOAPnuke (version 1.5.2) by removing reads with a sequencing adapter, low-quality base (>20%) or an unknown base (*N* >5%). Clean reads were then obtained and stored in FASTQ format. Clean reads were mapped to the reference genome with HISAT2 (version 2.0.4) and aligned to the reference coding gene set with Bowtie2 (version 2.2.5). Gene expression was quantified using RNA-Seq by Expectation Maximization (RSEM) (version 1.2.12), and differential expression analysis was conducted using DESeq2 (version 1.4.5) with a *Q* value of 0.05 or less. KEGG enrichment analysis was performed on annotated differentially expressed genes according to a hypergeometric test. The significance levels of terms and pathways were corrected for by Bonferroni test with a rigorous threshold (*Q* ≤ 0.05).

### Major cell-type clustering and marker gene identification.

The scRNA-Seq cells were categorized into 6 major subpopulations: epithelial cells, stromal cells, B cells, neutrophils, MoMϕDCs, and NKT cells. After refining the stromal, MoMϕDC, and NKT cell clusters, specific gene markers were identified using FindAllMarkers, and clusters were annotated on the basis of canonical marker gene expression. After reintegrating these 6 major subpopulations, we performed principal component analysis (PCA) on the list of highly variable genes using the RunPCA function in Seurat and visualized the clustering using uniform manifold approximation and projection (UMAP), ultimately identifying 18 distinct subclusters.

### Reconstructing cellular trajectories of tumor cells.

Different trajectory inference approaches were used to infer differentiation trajectories of tumor cells. RNA velocity analysis was performed using the velocyto R package (version 0.6) on unspliced and spliced matrices generated from the 10× scRNA-Seq BAM files. RNA velocity and plots were generated using the standard velocyto workflow. The CytoTRACE R package (version 0.3.3) was used to estimate the differentiation state of cells, with a score of 0 to 1 indicating stemness (higher score) or differentiation (lower score). Pseudotemporal analysis was performed using the Monocle 2 R package (version 2.24.0), with selection of genes expressed in more than 10 cells with a *Q* value of less than 0.0005 for analysis, followed by dimensionality reduction and trajectory construction using default methods.

### GSVA.

Pathway analysis was performed using KEGG gene sets from the MSigDB (version 7.2) with GSVA (GSVA package, version 1.44.2) under standard settings to assign pathway activity estimates to individual cells. Differential pathway activities between epithelial cell subclusters were obtained using the Limma package (version 3.52.2).

### Analysis of interaction between cell types.

To reveal the cell-cell interactions in the mouse ICC scRNA-Seq dataset, we used the Cellchat R package (version 1.4.0) to infer potential communication networks. Ligand-receptor pairs between epithelial cells and MoMϕDCs were mapped using the CellPhoneDB tool (version 2.1.7) with the default pipeline.

### Gene set enrichment analysis.

WNT scores were calculated for epithelial cells using the “AddModuleScore” function in Seurat, with the “WNT_SIGNALING” gene set from the C2 modules of MSigDB.

### Mouse-human ICC cell-type comparison Sankey diagram and heatmaps.

The publicly available human ICC single-cell HRA000863 dataset was used to compare mouse cell types and human ICC. Orthogonal genes were identified in both species, and mutual nearest neighbors were used to integrate cells, correcting the batch effects. The “RunFastMNN” function (SeuratWrappers, version 0.1.0) generated an integrated matrix after Seurat’s “sctransform” identified variable genes. Seurat was then used for cell-type identification and grouping. A Sankey diagram was created to visualize cell-type origins (35). Mouse and human ICC cell types were analyzed at single-cell and transcriptome levels, and dendrograms and heatmaps were made for comparisons across species (36).

### Histology, IHC, and multicolor IHC detection.

Mouse liver tissues were fixed in neutral formalin, embedded in paraffin, and sectioned at 5 μm. Sections were deparaffinized, rinsed in PBS, and subjected to antigen retrieval using sodium citrate antigen retrieval solution (C02-02002, Bioss). Endogenous peroxidase was blocked with 3% H_2_O_2_ (CS7730, G-clone) for 10 minutes, followed by blocking with 5% BSA for 30 minutes at 37°C. Sections were incubated overnight at 4°C with the following primary antibodies: HNF4A (ab41898, Abcam); MKI67 (NB500-170, Novus); cytokeratin 19 (ab52625, Abcam); RFP (600-401-379, Rockland); cleaved caspase 3 (9661S, Cell Signaling Technology [CST]); GAS6 (DF8659, Affinity); F4/80 (ab100790, Abcam); MRC1 (ab64693, Abcam); ATP6V1F (HPA062011, MilliporeSigma); SCP2 (MA5-44821, Thermo Fisher Scientific); AXL(AF7793, Affinity); and MERTK (ab300136, Abcam). Sections were then incubated with HRP-labeled secondary antibodies at 37°C for 30 minutes. Sections were stained with DAB, examined under a microscope, washed with PBS, and counterstained with hematoxylin, followed by dehydration, clearing, and mounting. Images were acquired using a digital section scanner (KF-PRO-020, KFBIO).

For immunofluorescence, sections were preincubated with 5% BSA for 30 minutes and then incubated with primary antibodies overnight at 4°C. HRP-labeled secondary antibodies were applied at room temperature for 15 minutes. Dyes 520, 570, and 650 (PANOVUE) were used to visualize antigen-binding sites. Images were captured using a confocal microscope (Zeiss LSM880 with Airyscan).

To assess cell proliferation in vivo, EdU (100 mg/mice) was injected i.p. and chased for 90 minutes. EdU incorporation was marked using a Click-iT EdU Alexa Fluor 488 Kit (C10337, Thermo Fisher Scientific) per the manufacturer’s protocol.

### Flow cytometric analysis and FACS.

Single-cell suspensions of mouse ICC samples were prepared as described above. Cells were washed in staining buffer (2% bovine growth serum in PBS) and resuspended to 1 × 10^7^ cells/mL. For extracellular staining, cells were stained with antibodies in the staining buffer for 1 hour at 4°C. For intracellular staining, cells were fixed for 45 minutes using the Foxp3/Transcription Factor Staining Buffer kit (Tonbo Biosciences, TNB-0607-KIT), and then washed and stained overnight at 4°C with antibodies in 1× permeabilization buffer. The following antibodies and dyes were used for flow cytometry: CD45 PE-Cyanine 7 (60-0451, Tonbo Biosciences); CD3 APC (20-0032, Tonbo Biosciences); CD8a VioletFluo 450 (75-1886, Tonbo Biosciences); CD4 FITC (35-0042, eBioscience); FOXP3 PerCP/Cy5.5 (55-5773-U100, Tonbo Biosciences); CD25 PE (50-0251-U100, Tonbo Biosciences); CD206 APC (141708, BioLegend), F4/80 PE (137014, BioLegend); F4/80 PerCP/Cy5.5 (2349828, eBioscience); CD86 PerCP/Cy5.5 (105028, BioLegend); NK1.1 PerCP/Cy5.5 (65-5941, Tonbo Biosciences); CD11b Violet Fluorescence 500 (85-0112, Tonbo Biosciences); Ghost Dye Red 780 (13-0865, Tonbo Bioscience); EpCAM APC (B358247, Invitrogen, Thermo Fisher Scientific); AXL BV421 (748028, BD Biosciences); Ghost Dye Red 710 (13-0871-T100, Tonbo Biosciences); MERTK APC-CY7 (47-5751-80, Invitrogen, Thermo Fisher Scientific); GAS6 FITC (bs-7549R-BF488, Bioss); Siglec APC (129611, BioLegend); MHC II FITC (35-5321-U100, Tonbo Biosciences); CD11C Violet Fluo 450 (75-0114-U025, Tonbo Biosciences); LY6G APC-CY7 (127623, BioLegend); and LY6C (45-5932-80, Invitrogen, Thermo Fisher Scientific). Samples were analyzed using a flow cytometer (CytoFLEX, Beckman Coulter), and data were analyzed with NovoExpress software (version 2.0).

To sort intratumoral Reg-TAMs, single-cell suspensions of digested tumors were stained with Ghost Dye Red 780 for 30 minutes, followed by 500 ng Fc blocker (anti-CD16/anti-CD32, Elabscience) for 15 minutes and then with anti-CD45 PE-Cyanine 7, CD11b Violet 500, F4/80 PE, and CD206 APC (all from BioLegend) in 1 mL staining buffer (PBS plus 2% FBS) for 30 minutes at 4°C. Live CD206^+^ macrophages were sorted on a FACSAria Fusion flow cytometer (BD).

### Limiting dilution assay.

For limiting dilution transplantation in nude mice, FACS-sorted cells (EpCAM^+^Tom^+^ or EpCAM^+^Tom^–^) from primary ICCs from *Krt19-DreER* *Axin2-CreER* *R26-Ai66* mice were used. Five-week-old BALB/c-nu/nu nude mice were anesthetized with 87.5 mg/kg ketamine and 12.5 mg/kg xylazine and s.c. injected with the cells in the dorsal region. Mice were then placed in a sterile laminar flow chamber and monitored for 24 hours for vital signs.

### Human tissue collection.

ICC tissues and adjacent tissues were collected from patients who underwent surgery at the FAHSYSU or Sun Yat-sen University Cancer Center. Written informed consent was obtained from all patients prior to the tissue collection.

### Western blotting.

To detect the protein expression of Reg-TAMs, Reg-TAMs were isolated and sorted from tumors as previously described and then lysed in ice-cold RIPA buffer (P0013B, Beyotime) with protease and phosphatase inhibitors (4693132001, Roche) using a gentleMACS Dissociator (130-093-235, Miltenyi Biotec). For mouse liver tissue, tissues were dissected and lysed in the same buffer. The protein concentration was measured using the BCA assay (23227, Thermo Fisher Scientific). Equal protein amounts were separated by 10% SDS-PAGE (PG11X, EpiZyme), transferred onto PVDF membranes (IPVH00010, MilliporeSigma), and probed with antibodies against AXL (ab215205, Abcam); p-AXL (AF8523, Affinity); MERTK (ab300136, Abcam); p-MERTK (PA5-143631, Thermo Fisher Scientific); GAS6 (DF8659, Affinity); β-catenin (ab32572, Abcam); p–β-catenin (9561, CST); LRP6 (3395, CST); p-LRP6 (2568, CST); CCL8 (55062, Signalway Antibody [SAB]); GAPDH (2118S, CST); NF-κB1 (13586, CST); AKT (9272, CST); and p-AKT (9271, CST). After washing, the blots were incubated with an HRP-conjugated secondary antibody (SA00001-2, Proteintech), and bands were visualized using ECL (180-5001, Tanon).

### Treg differentiation assay.

Naive CD4^+^ T cells were isolated from C57BL/6 mice using the EasySep Mouse Naive CD4^+^ T Cell Isolation Kit (19765, STEMCELL Technologies). Reg-TAMs were sorted by flow cytometry and cocultured with naive CD4^+^ T cells in lymphocyte culture medium (88-581-cm, Corning) at 37°C in 5% CO_2_ for 3 days. IL-2 (10 ng/mL, PKSM041320, Elabscience) and TGF-β1 (10 ng/mL, PRP110618, Abbkine) were added to promote Treg differentiation. After 72 hours, cells were analyzed by flow cytometry for FOXP3 and CD25 expression to assess Treg differentiation.

### Proliferation assay for CD8^+^ T cells.

To assess the effect of Reg-TAMs on CD8^+^ T cell proliferation, CD8^+^ T cells were isolated from ICC-bearing C57BL/6 mice using flow cytometry, and Reg-TAMs were sorted from ICC tumors with or without *Axl* and *Mertk* KO. The CD8^+^ T cells were resuspended in PBS (1 × 10^7^ cells/mL) and labeled with 5 μM CFSE (565082, Biosciences) for 10 minutes at room temperature and then quenched with lymphocyte medium containing10% FBS. After washing, Reg-TAMs were added to the coculture in a medium with 10% FBS, and IL-2 (10 ng/mL, PKSM041320, Elabscience). Cells were incubated at 37°C and 5% CO_2_ for 72 hours and then analyzed by flow cytometry for CD8^+^ T cell proliferation.

### ELISA.

CCL8 concentrations in Reg-TAMs of ICC tumors were measured using ELISA kits (JL10984, JONLN) according to the manufacturer’s instructions. Briefly, 100 μL samples were incubated on 96-well plates at 37°C for 1 hour, followed by biotinylated primary antibody incubation for 1 hour. After washing, streptavidin-HRP was added and incubated for 30 minutes and then washed, and TMB substrate was added for 15 minutes in the dark. Absorbance was measured using a TECAN Infinite M200Pro reader.

### RNA extraction and qRT-PCR analysis.

Total RNA from sorted macrophages was extracted with AG RNAex Pro Reagent (Accurate Biotechnology, AG21102) according to the manufacturer’s instructions. The RNA concentration was measured using a NanoDrop spectrophotometer (Nano-300, Thermo Fisher Scientific). qRT-PCR was performed using PerfectStart Green qPCR SuperMix (TransGen Biotech, AQ601) on a Bio-Rad CXF96 real-time system. The relative quantity was calculated using an internal control.

### Statistics.

In this study, numerical data and histograms are presented as the mean ± SD. Statistical parameters are provided in the figure legends. All experiments were performed at least twice. The data shown are representative or a combination of independent experiments. A 2-tailed, unpaired Student’s *t* test was used for comparisons between 2 groups. For multiple-group comparisons (*n* >2), 1-way ANOVA was used. Survival analysis was performed with the log-rank test. Limiting dilutions were analyzed using extreme limiting dilution analysis (ELDA) software (http://bioinf.wehi.edu.au/software/elda/). A *P* value of less than 0.05 was considered significant. All statistical analyses were conducted using GraphPad Prism 9 (GraphPad Software).

### Study approval.

All animal experiments were approved by the IACUC of Sun Yat-Sen University (protocol number SYSU-IACUC-2021-000138, Guangzhou, China). All patients provided written informed consent prior to undergoing surgical treatment.

### Data availability.

The raw sequencing data reported in this work (including scRNA-Seq and bulk RNA-Seq data) have been deposited in the Genome Sequence Archive (GSA) at the National Genomics Data Center (accession numbers CRA008695 and CRA008863). The analysis of public datasets was retrieved from GSA HRA000863 (8). The remaining data can be found in the article, supplemental information, or in the Supporting Data Values file. This study did not use any custom computer code or algorithms.

## Author contributions 

## SC, MC, KL, XW, and DC conceptualized the study. SC, GT, RZ, MC, RL, CZ, ZZ, GX, and DC designed the study methodology. SC, KL, MC, YZ, ZZ, and DC curated and analyzed the data. SC, KL, MC, YZ, YL, ZZ, and DC, JM, and JX performed experiments and validated the results. CH, BZ, ZP, LP, DC, and HL provided resources. SC, KL, and DC wrote the manuscript. LP and DC supervised the study and acquired funding.

## Supplementary Material

Supplemental data

Unedited blot and gel images

Supplemental table 1

Supplemental table 2

Supplemental table 3

Supporting data values

## Version 1 01/07/2025

In-Press Preview

## Version 2 03/03/2025

Electronic publication

## Version 3 07/16/2026

Updated numerical precision and percentage formatting in Supporting Data Value file
