# Supplementary material for: Tumor-initiating cells escape tumor immunity via CCL8 from tumor-associated macrophages in mice
Source: J Clin Invest. 2025 Jan 7;135(5):e180893. doi: 10.1172/JCI180893 (PMC11870738; doi:10.1172/JCI180893)
Supplement: Supplemental data [file jci-135-180893-s008.pdf]

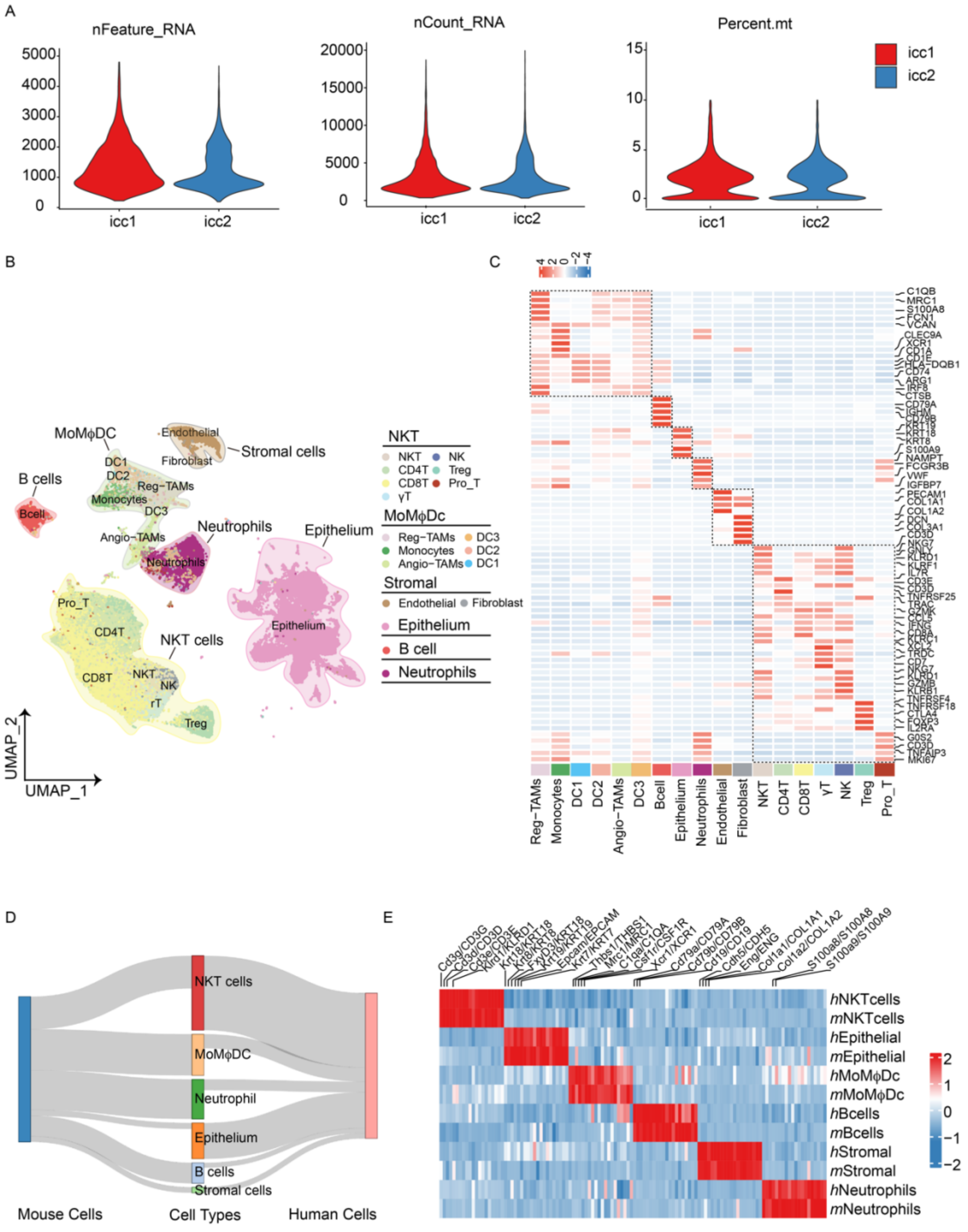

Figure S1, Related to Figure 1. Mouse and human ICC contain a similar cellular composition and conserved gene signatures within the same cell type

A. Violin plots of the number of genes, total number of RNA reads, and percentage of mitochondrial genes per cell in each sample.

B. UMAP of single-cell clusters from human ICC tumor tissues, colored by cell cluster.

C. Heatmap of signature genes for eighteen cell clusters.

D. Sankey diagram represents the contribution of the mouse cells and human cells to the six major groups.

E. Heatmap of six major groups showing genes similarly enriched within mouse and human cell clusters from mice ICC tumor tissues and human ICC tumor tissues.

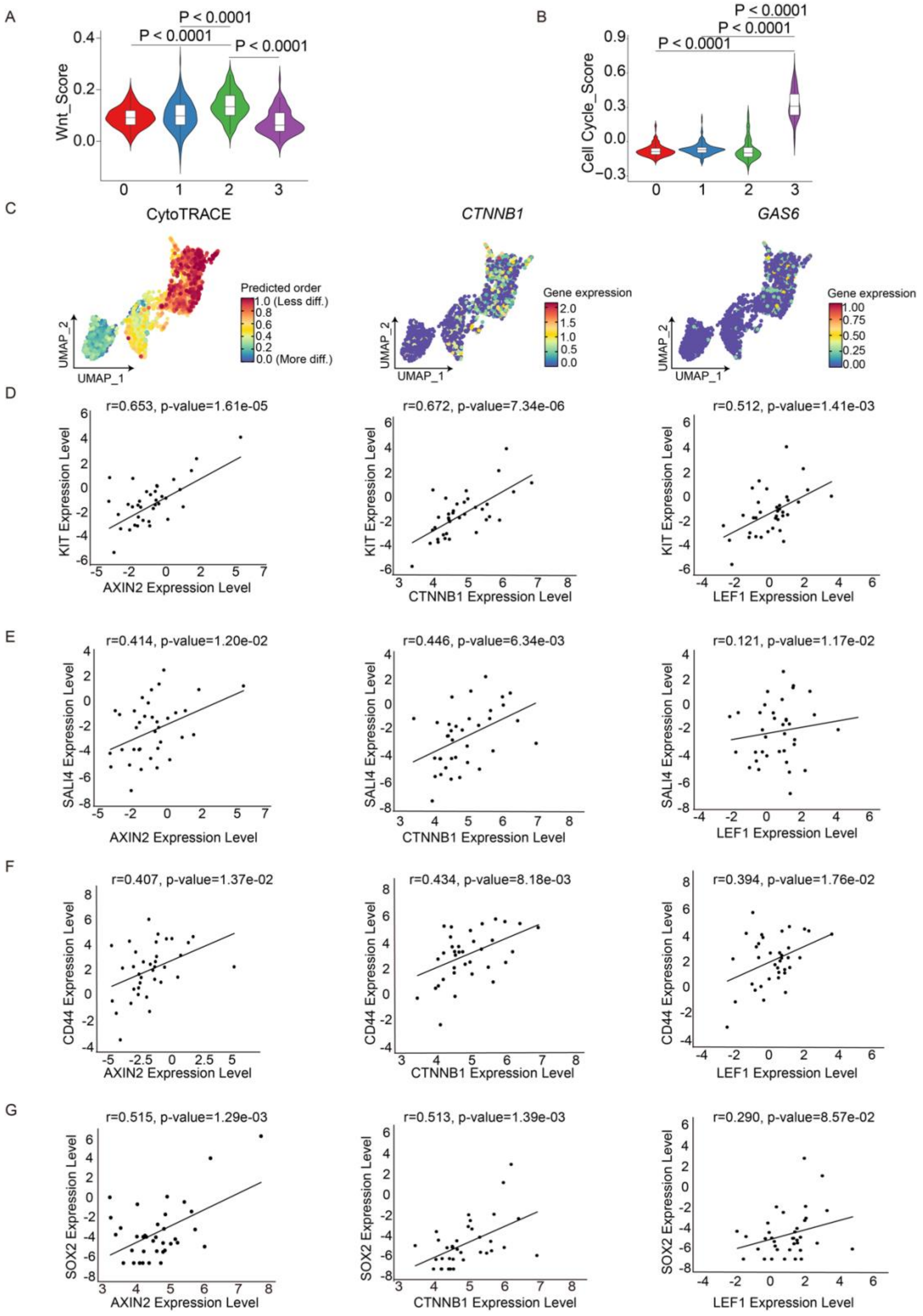

Figure S2, Related to Figure 2. WNT/β-catenin signaling is enriched in the TICs of mouse and human ICC samples

A. Violin plot depicts WNT pathway score among four epithelial subclusters. *P* values

were calculated by a non-parametric Wilcoxon test.

B. Violin plot depicts cell cycle pathway score among four epithelial subclusters. *P* values were calculated by non-parametric Wilcoxon test.

C. UMAP plots showing the CytoTRACE scores distribution among the epithelial cells from one representative human ICC sample (left). Feature plots of *CTNNB1* (middle) and *GAS6* (right) gene expression in the epithelial cells in the human ICC sample.

D-G. Correlation between *KIT* (D), *SALL4* (E), *CD44* (F), *SOX2* (G), and canonical WNT/β-catenin signaling pathway genes (*AXIN2*, *CTNNB1*, and *LEF1*) were analyzed by Pearson's correlation test according to the TCGA-CHOL datasets.

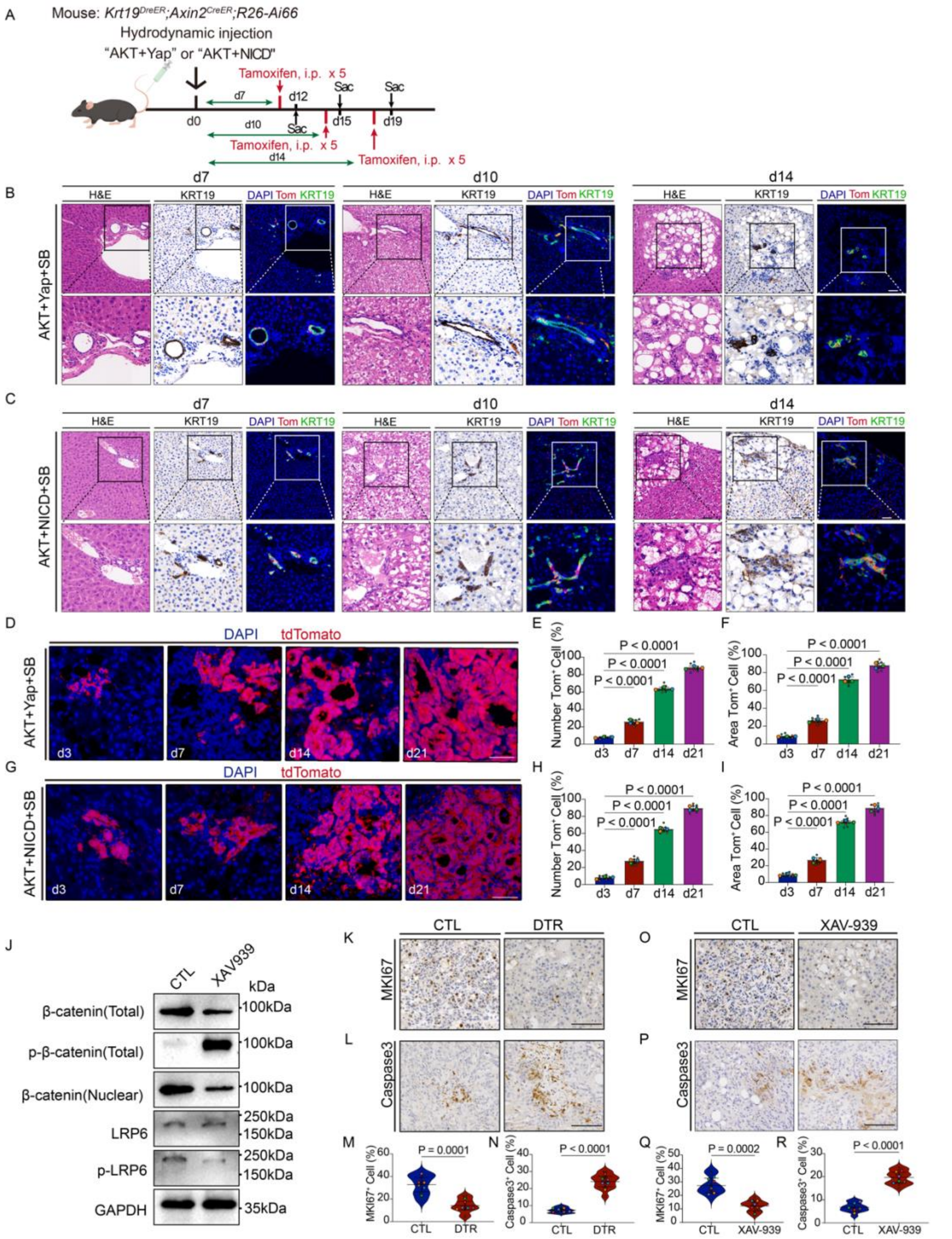

Figure S3, Related to Figure 2. WNT-activated cells and WNT/β-catenin signaling are responsible for mouse ICC progression

A. Experimental strategies for lineage-tracing of KRT19^{+} AXIN2^{+} cells of ICC in *Krt19-DreER; Axin2-CreER; R26-Ai66-DTR* mice. KRT19^{+} AXIN2^{+} cells were labeled after tamoxifen were given.

B-C. Representative images of H&E staining, KRT19 IHC staining, and KRT19 and RFP IF staining at days 7, 10, and 14 in ICC after AKT/YAP/SB treatment (B) and AKT/NICD/SB treatment (C). Nuclei are stained with DAPI (blue). Scale bar, 50 μm.

D-I. Fluorescence images of lineage tracing in ICC induced by AKT/YAP/SB (D) and AKT/NICD/SB (G) after tamoxifen treatment. Nuclei are stained with DAPI (blue). Scale bar, 50 μm. Comparisons of the number (E, H) and area (F, I) of Tom^+ cells in ICC for 3, 7, 14, and 21 days after AKT/YAP/SB or AKT/NICD/SB treatment. Error bars represent mean ± SD from three independent experiments (n = 9 fields from 3 mice). *P* values were calculated by one-way ANOVA with Tukey's multiple comparison test.

J. Western blotting analysis of β-catenin (Total), p-β-catenin (Total), β-catenin (Nuclear), LRP6, p-LRP6, and GAPDH in ICC TICs after XAV-939 treatment.

K and L. Representative image of MKI67 (K) and active-Caspase3 (L) staining of liver sections from ICC-induced mice after diphtheria toxin treatment. Scale bar, 100μm.

M and N. Statistical analysis of $MKI67^+$ (M) and active-$Caspase3^+$ (N) cells after diphtheria toxin treatment. Values are mean ± SD from six independent biological replicates (n = 12 fields from 6 mice). *P* values were calculated by two-tailed unpaired Student's t-test.

O and P. Representative image of MKI67 (O) and active-Caspase3 (P) staining of liver sections from ICC-induced mice after XAV-939 treatment. Scale bar, 100μm.

Q and R. Statistical analysis of $MKI67^+$ (Q) and active-$Caspase3^+$ (R) cells after XAV-939 treatment. Values are mean ± SD from six independent biological replicates (n = 12 fields from 6 mice). *P* values were calculated by two-tailed unpaired Student's t-test.

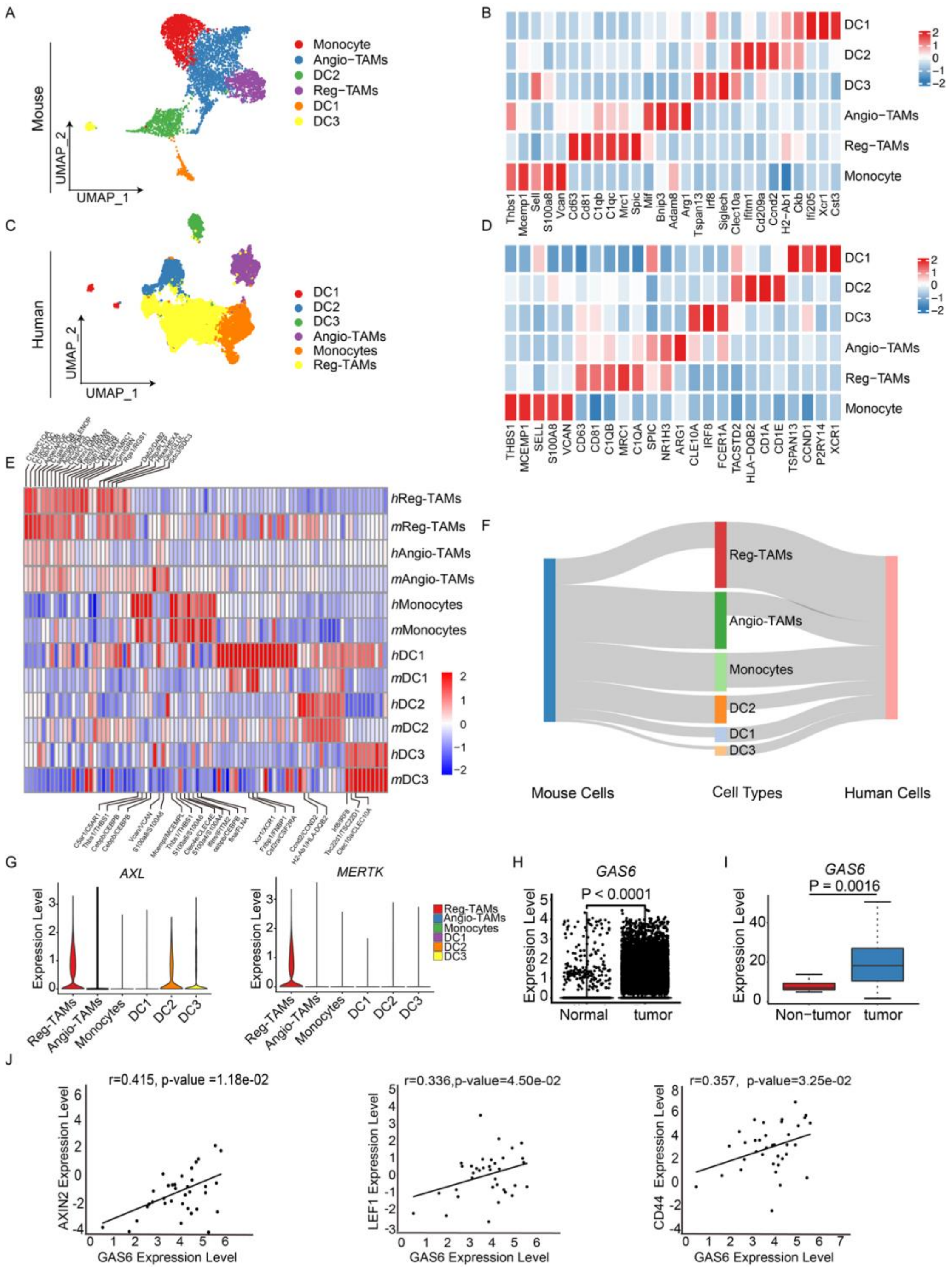

Figure S4, Related to Figure 4. Expression of GAS6-AXL/MERTK in human ICC samples

A-D. UMAP of myeloid cell clusters from mouse (A) and human (C) ICC tumor tissues, colored by cell cluster. Heatmap of signature genes for myeloid cell clusters from mouse (B) and human (D) ICC tumor tissues, with each cell cluster represented by

specifically expressed genes.

E. Heatmap showing genes similarly enriched within mouse and human myeloid cell clusters in ICC tumor tissues.

F. Sankey diagram represents the contribution of the mouse cells and human cells to the myeloid cell clusters.

G. Violin plot depicts the expression of *AXL* and *MERTK* in myeloid cell clusters from human ICC tumor tissues.

H. Violin plot depicts expression of *GAS6* in normal and ICC tumor cells according to human ICC scRNAseq datasets. *P* value was calculated by two-tailed unpaired Student’s t-test.

I. Expression of *GAS6* mRNA is higher in human non-tumor control compared with ICC tissues according to the TCGA-CHOL datasets. *P* value was calculated by two-tailed unpaired Student’s t-test.

J. Correlations between *AXIN2*, *LEF1*, *CD44*, and *GAS6* mRNA expression were analyzed by Pearson's correlation test according to the TCGA-CHOL datasets.

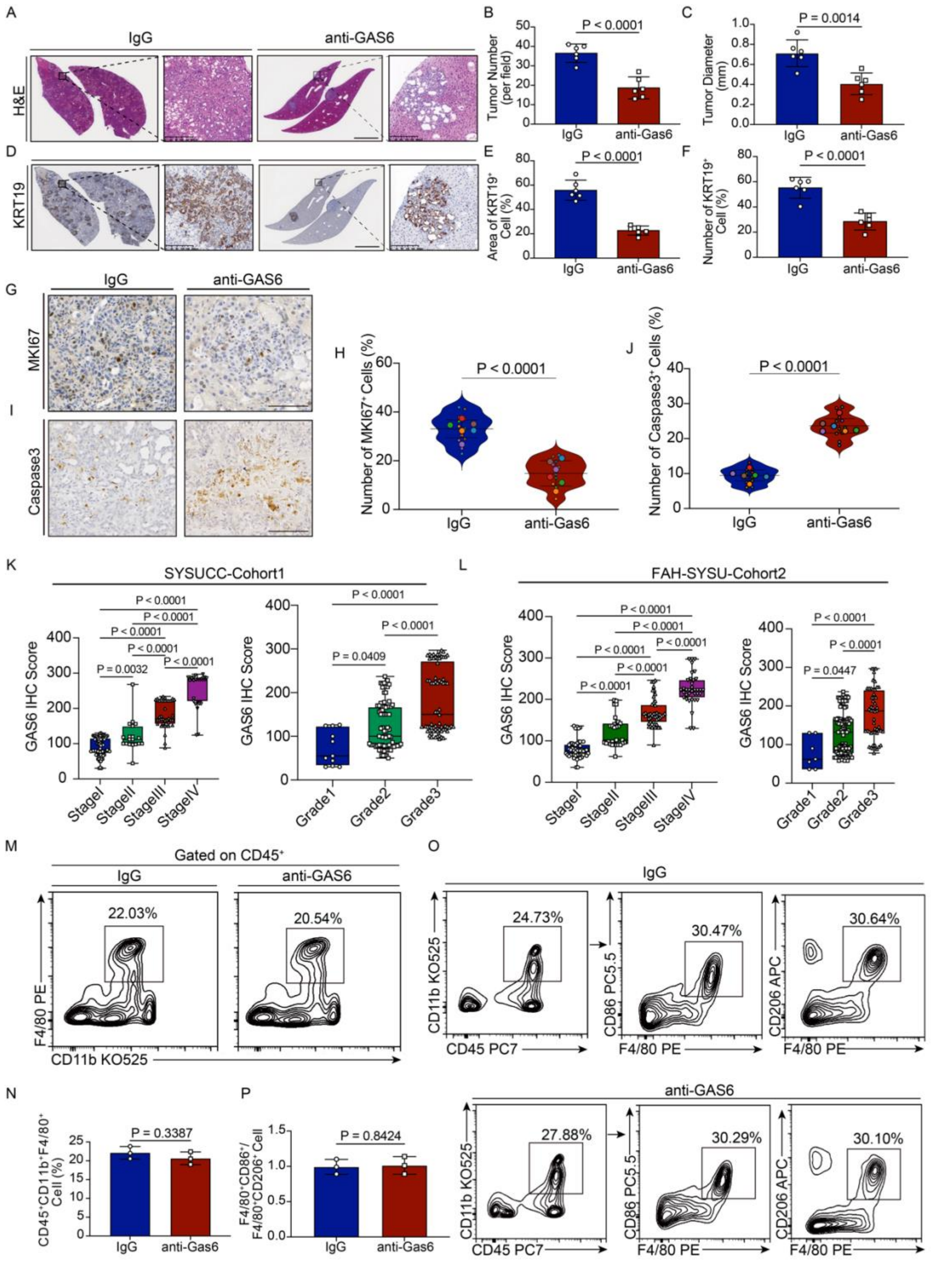

Figure S5 Related to Figure 4. Blocking of GAS6 led to inhibition of ICC development A-F. Representative images of H&E staining (A) and KRT19 staining (D) of liver sections in ICC mice treated with anti-GAS6 or IgG, with scale bars of 200 μm. Statistical analyses include ICC number (B), ICC diameter (C), KRT19 area (E), and

KRT19$^+$ cells (F) in the anti-GAS6 and IgG groups. Values are mean ± SD from six independent biological replicates (n = 6 mice). *P* values were calculated by two-tailed unpaired Student's t-test.

G-J. Representative images of MKI67 (G) and active-Caspase3 (I) staining of liver sections and their respective statistical analyses of MKI67$^+$ cells (H) and active-Caspase3$^+$ cells (J) in the anti-GAS6 and IgG groups. Scale bars, 100 μm. Values are mean ± SD from six independent biological replicates (n = 12 fields from 6 mice). *P* values were calculated by two-tailed unpaired Student's t-test.

K. GAS6 IHC score in different tumor stages (left) and tumor grades (right) from SYSUCC cohort1. *P* values were calculated by one-way ANOVA with Tukey's multiple comparison test.

L. GAS6 IHC score in different tumor stages (left) and tumor grades (right) from FAH-SYSU cohort2. *P* values were calculated by one-way ANOVA with Tukey's multiple comparison test.

M-P. In a multi-color DC profiling panel, SSC and FSC parameters were used to isolate mononuclear cells, and CD45$^+$ mononuclear cells were confirmed as immune cells. Macrophages were defined as CD11b$^+$F4/80$^+$, M1 macrophages identified as F4/80$^+$CD86$^+$ and M2 macrophages as F4/80$^+$CD206$^+$. Representative flow plots show the frequency of these specific cell clusters in the anti-GAS6 and IgG groups (M, O). Quantification includes CD11b$^+$F4/80$^+$ cells (N) and the ratios of F4/80$^+$CD86$^+$/F4/80$^+$CD206$^+$ cells (P) in different groups. Values are mean ± SD from three independent experiments (n = 3 mice). *P* values were calculated by two-tailed unpaired Student's t-test.

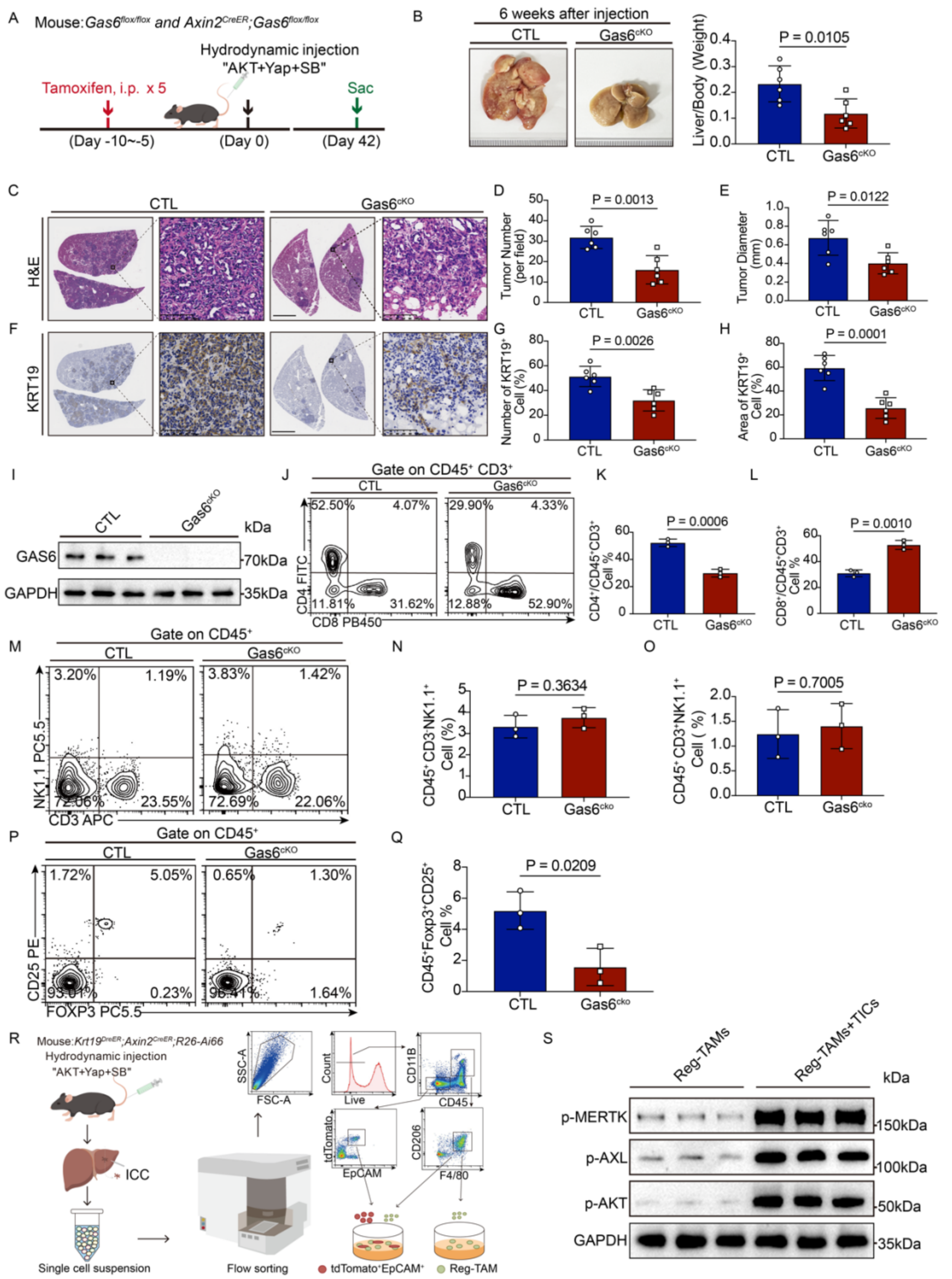

Figure S6. Related to Figure 4. Epithelial-specific knockout of GAS6 leads to the inhibition of ICC development

A. Experimental design for induction of ICC in *Gas6*$^{flox/flox}$ (Control group) and *Axin2-creER; Gas6*$^{flox/flox}$ (Gas6^{cko} group) mice. The mice were sacrificed six weeks after the plasmids were injected.

B. Representative image of whole liver morphology in control and Gas6^{cKO} mice with the plasmid injected for six weeks (left). Statistical analysis of liver to body weight ratio in control and Gas6^{cKO} mice (right). Values are mean ± SD from six independent biological replicates (n = 6 mice). *P* value was calculated by two-tailed unpaired Student's t-test.

C-H. Representative images of H&E staining (C) and KRT19 staining (F) of liver sections in control and Gas6^{cKO} groups, with scale bars of 100 μm. Statistical analysis of ICC number (D), ICC diameter (E), KRT19^{+} cells (G), and KRT19^{+} area (H) in control and Gas6^{cKO} groups. Values are mean ± SD from six independent biological replicates (n = 6 mice). *P* values were calculated by two-tailed unpaired Student's t-test.

I. The knockout efficiency of GAS6 in Axin2^{+} cells was validated by WB analysis.

J-L. Representative flow plots showing the frequency of CD4^{+} and CD8^{+} T cells in control and Gas6cKO mice (J). Statistical analysis of the number of CD4^{+} (K) and CD8^{+} (L) T cells in control and Gas6^{cKO} mice. Values are mean ± SD from three independent experiments (n = 3 mice). *P* values were calculated by two-tailed unpaired Student's t-test.

M-O. Representative flow plots showing the frequency of NK and NKT cells in control and Gas6^{cKO} mice (M). Statistical analysis of the number of NK cells (N) and NKT cells (O) in control and Gas6^{cKO} mice. Values are mean ± SD from three independent experiments (n = 3 mice). *P* values were calculated by two-tailed unpaired Student's t-test.

P-Q. Representative flow plots showing the frequency of CD25^{+} Foxp3^{+} cells in control and Gas6^{cKO} mice (P). Statistical analysis of the number of CD25^{+} Foxp3^{+} cells in control and Gas6^{cKO} mice (Q). Values are mean ± SD from three independent experiments (n = 3 mice). *P* value was calculated by two-tailed unpaired Student's t-test.

R. The schematic diagram illustrating the sorting and co-culture of TICs and Reg-TAM cells.

S. Western blot analysis of phosphorylated AXL, MERTK, AKT, and GAPDH in ICC Reg-TAMs co-cultured with or without TICs treatments.

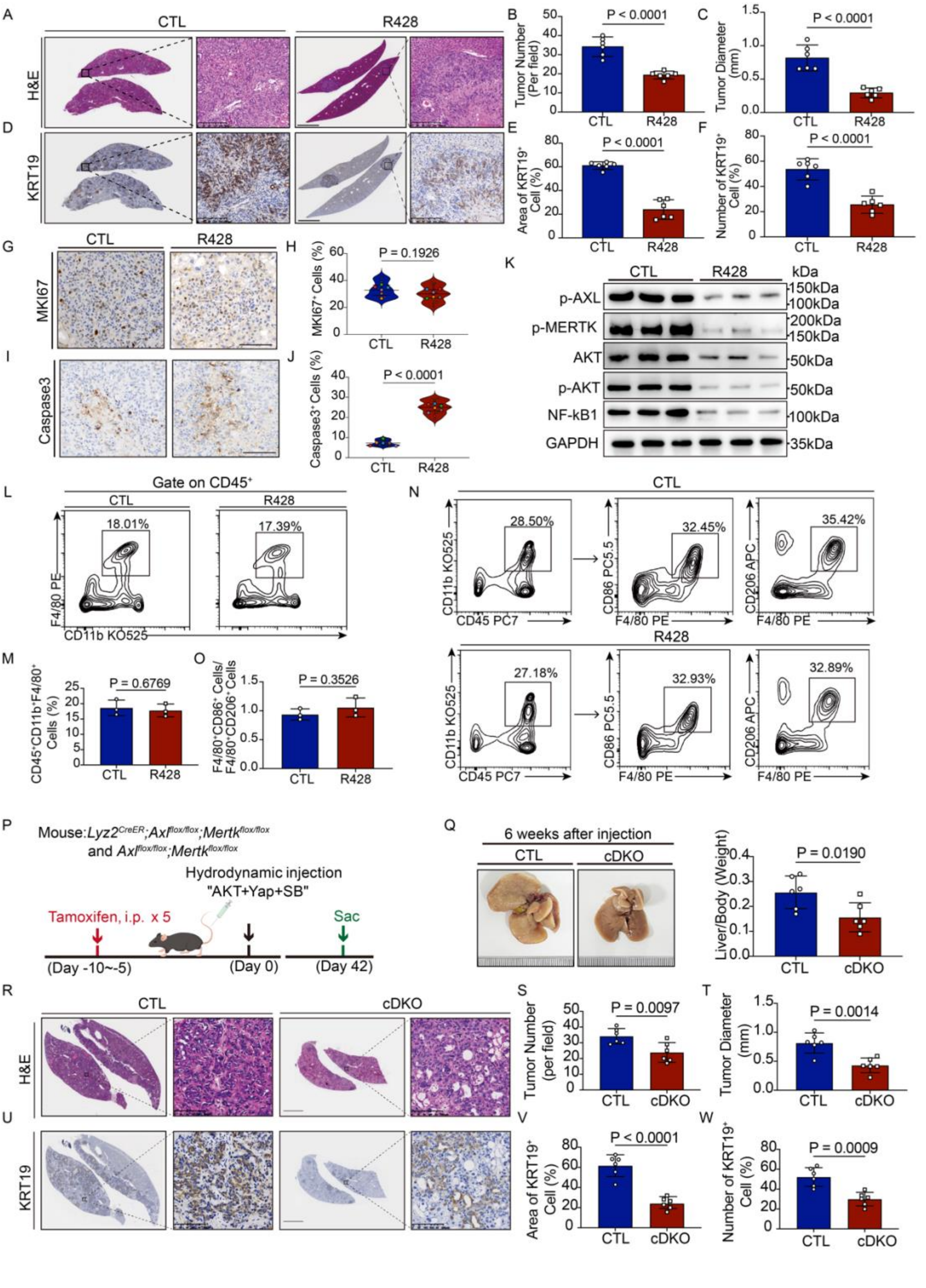

Figure S7 Related to Figure 5. Treatment with R428 or double knockout of *Axl* and *Mertk* inhibited ICC formation

A-F. Representative images of H&E staining (A) and KRT19 staining (D) of liver sections in ICC mice from R428 treatment or control groups. Scale bar, 200 μm.

Statistical analysis of ICC number (B), ICC diameter (C), KRT19 area (E), and KRT19$^+$ cells (F) in different groups. Values are mean ± SD from six independent biological replicates (n = 6 mice). *P* values were calculated by two-tailed unpaired Student's t-test.

G-J. Representative images of MKI67 (G) and active-Caspase3 (I) staining of liver sections in the R428 treatment or control groups. Scale bar, 100 μm. Statistical analysis of MKI67$^+$ (H) and active-Caspase3$^+$ (J) cells in the same groups. Values are mean ± SD from six independent biological replicates (n = 12 fields from 6 mice). *P* values were calculated by two-tailed unpaired Student's t-test.

K. Western blot analysis of p-AXL, p-MERTK, p-AKT, AKT, NF-κB1, and GAPDH in Reg-TAMs with or without R428 treatment, after co-culturing with EpCAM$^+$ Tom$^+$ TICs.

L and M. Representative flow plots show the frequency of the CD11b$^+$ F4/80$^+$ macrophages as indicated in the control and R428 group (L). Quantification of the CD11b$^+$ F4/80$^+$ cells in control and R428 group (M). Values are mean ± SD (n = 3 mice). *P* value was calculated by two-tailed unpaired Student's t-test.

N and O. Representative flow plots show the frequency of the F4/80$^+$CD86$^+$ macrophages and F4/80$^+$CD206$^+$ macrophages as indicated in the control and R428 group (N). Quantification of the ratios of F4/80$^+$CD86$^+$/ F4/80$^+$CD206$^+$ cells in WT and R428 group (O). *P* value was calculated by two-tailed unpaired Student's t-test.

P. Experimental design for induction of ICC in *Axl$^{flox/flox}$; Mertk$^{flox/flox}$* (Control group, CTL) and *Lyz2-CreER; Axl$^{flox/flox}$; Mertk$^{flox/flox}$* (*Axl* and *Mertk* conditional double knockout, cDKO group) mice. The mice were sacrificed six weeks after the plasmids were injected.

Q. Representative image of whole liver morphology in CTL and cDKO mice with the plasmid injected for six weeks (left). Statistical analysis of liver to body weight ratio in CTL and cDKO mice (right). Values are mean ± SD from six independent biological replicates (n = 6 mice). *P* value was calculated by two-tailed unpaired Student's t-test.

R-W. Representative images of H&E (R) and KRT19 (U) staining of liver sections in different treatment groups, with scale bars of 100 μm. Statistical analysis of ICC number (S), ICC diameter (T), KRT19$^+$ area (V), and KRT19$^+$ cells (W) in CTL and

cDKO groups. Values are mean ± SD from six independent biological replicates (n = 6 mice). *P* values were calculated by two-tailed unpaired Student's t-test.

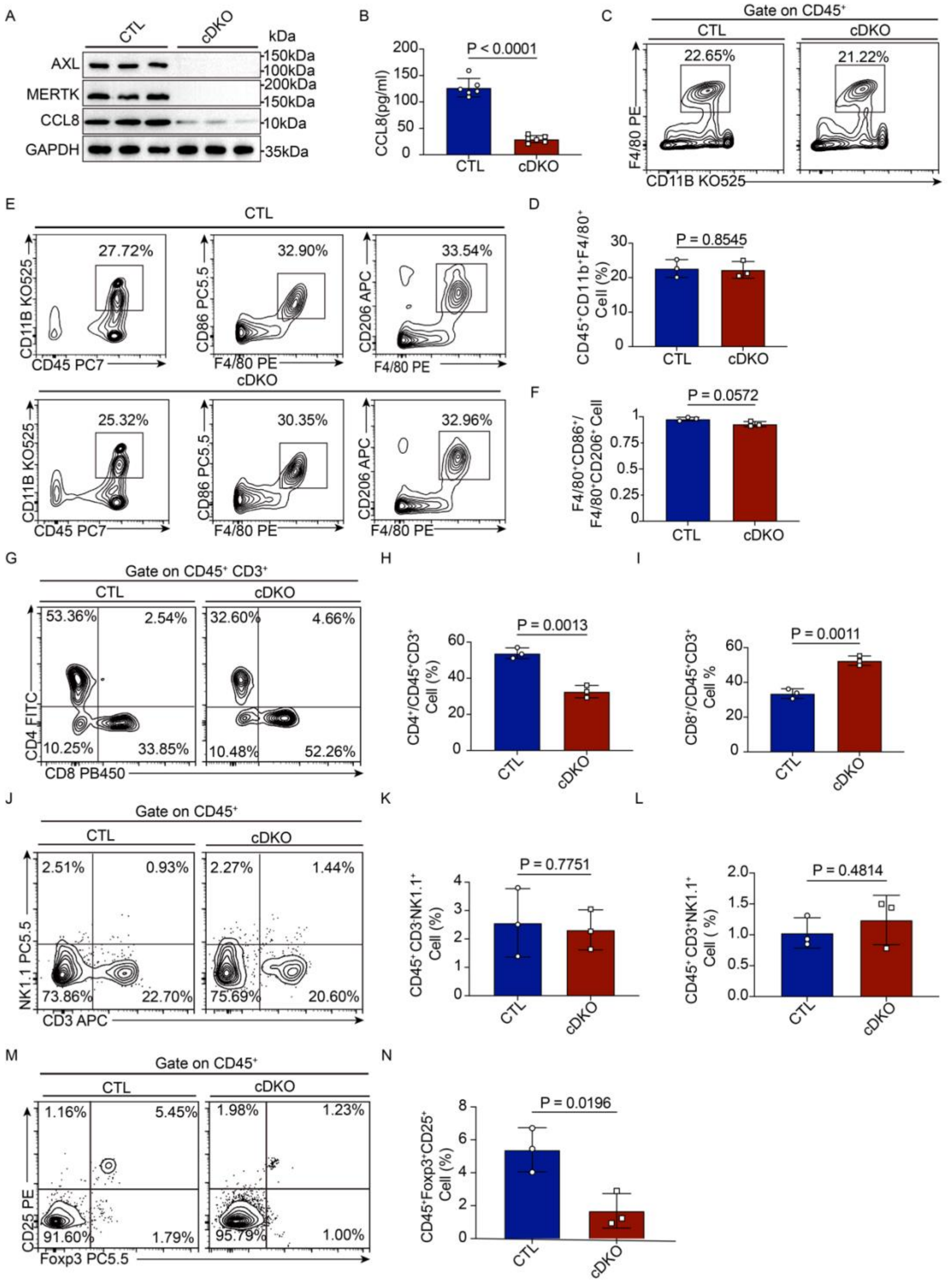

Figure S8 Related to Figure 5. Conditional knockout of *Axl* and *Mertk* inhibited ICC formation.

A. Western blotting analysis of AXL, MERTK, CCL8, and GAPDH in ICC Reg-TAMs in Control (CTL group) and *Lyz2-CreER; Axl$^{flox/flox}$; Mertk$^{flox/flox}$* (*Axl* and *Mertk* conditional double knockout, cDKO group) mice.

B. ELISA showed the protein levels of CCL8 in Reg-TAMs between CTL and cDKO groups. Values are mean ± SD from six independent biological replicates (n = 6 mice). *P* value was calculated by two-tailed unpaired Student's t-test.

C and D. Representative flow plots show the CD11b$^+$F4/80$^+$ macrophage frequency as indicated in CTL and cDKO groups (C). Quantification of the CD11b$^+$F4/80$^+$ cells in CTL and cDKO groups (D). Values are mean ± SD (n = 3 mice). *P* value was calculated by two-tailed unpaired Student's t-test.

E and F. Representative flow plots show the frequency of the F4/80$^+$CD86$^+$ macrophages and F4/80$^+$CD206$^+$ macrophages as indicated in CTL and cDKO groups (E). Quantification of the ratios of F4/80$^+$CD86$^+$/ F4/80$^+$CD206$^+$ cells in CTL and cDKO groups (F). *P* value was calculated by two-tailed unpaired Student's t-test.

G-I. Representative flow plot showing the frequency of CD4$^+$ and CD8$^+$ T cells in CTL and cDKO mice (G). Statistical analysis of the number of CD4$^+$ (H) and CD8$^+$ (I) T cells in CTL and cDKO mice. Values are mean ± SD from three independent experiments (n = 3 mice). *P* values were calculated by two-tailed unpaired Student's t-test.

J-L. Representative flow plot showing the frequency of NK and NKT cells in CTL and cDKO mice (J). Statistical analysis of the number of NK (K) and NKT (L) cells in CTL and cDKO mice. Values are mean ± SD from three independent experiments (n = 3 mice). *P* values were calculated by two-tailed unpaired Student's t-test.

M and N. Representative flow plot showing the frequency of CD25$^+$ Foxp3$^+$ cells in CTL and cDKO mice. Statistical analysis of the number of CD25$^+$ Foxp3$^+$ cells in CTL and cDKO mice. Values are mean ± SD from three independent experiments (n = 3 mice). *P* value was calculated by two-tailed unpaired Student's t test.

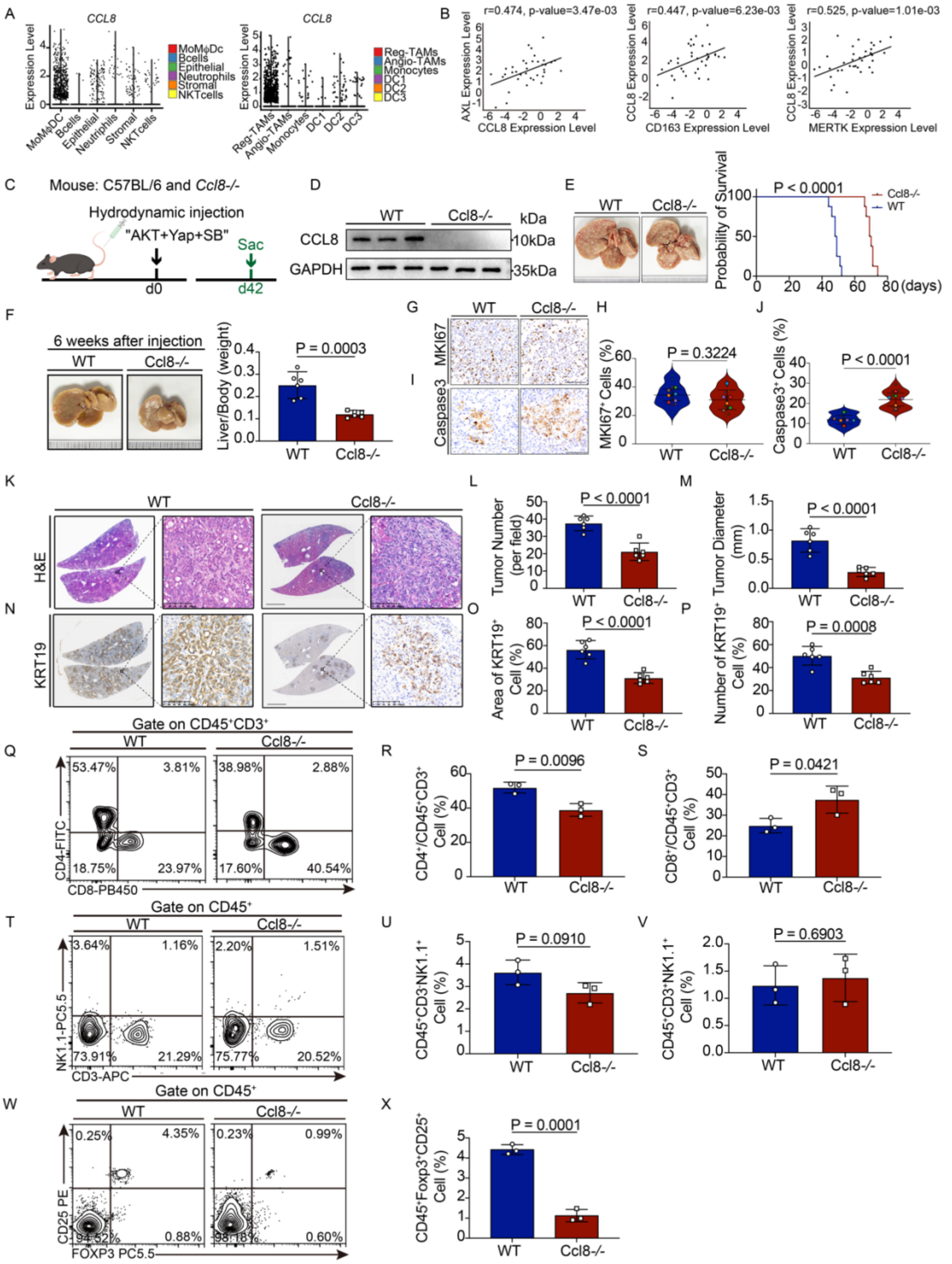

Figure S9 Related to Figure 6. CCL8 is the downstream mediator of AXL signaling in Reg-TAMs

A. Violin plots showed expression of *AXL* in all six clusters (left) and MoMϕDC clusters (right) from human ICC tumor tissues.

B. Correlation between *AXL*, *CD163*, *MERTK*, and *CCL8* expression were analyzed by

Pearson's correlation test according to the TCGA-CHOL datasets.

C. Experimental design for induction of ICC in C57BL/6 (WT group) and *Ccl8-/-* (*Ccl8-/-* group) mice.

D. The knockout efficiency of *Ccl8* was validated by WB analysis.

E. Representative image of whole liver morphology in WT and *Ccl8-/-* KO mice (left). The time of mice developed lethal tumor burden was shown. The Kaplan-Meier overall survival curve of WT and *Ccl8-/-* mice (right). *P* value was calculated by log-rank test.

F. Representative image of whole liver morphology in WT and *Ccl8*-/- mice with the plasmid injected for six weeks (left). Statistical analysis of liver to body weight ratio in WT and *Ccl8-/-* mice (right). Values are mean ± SD from six independent biological replicates (n = 6 mice). *P* value was calculated by two-tailed unpaired Student's t-test.

G-J. Representative images of MKI67 (G) and active-Caspase3 (I) staining of liver sections from ICC mice in WT and *Ccl8-/-* groups, with scale bars of 100 μm. Statistical analysis of MKI67^{+} (H) and active-Caspase3^{+} cells (J) in WT and *Ccl8-/-* groups. Values are mean ± SD from six independent biological replicates (n = 12 fields from 6 mice). *P* values were calculated by two-tailed unpaired Student's t-test.

K-P. Representative images of H&E staining (K) and KRT19 staining (N) of liver sections from ICC mice in WT and *Ccl8-/-* groups, with scale bars of 100 μm. Statistical analyses include ICC number (L), ICC diameter (M), KRT19 area (O), and KRT19^{+} cells (P) in WT and *Ccl8-/-*groups. Values represent mean ± SD from six independent biological replicates (n = 6 mice). *P* values were calculated by two-tailed unpaired Student's t-test.

Q-S. Representative flow plots showing the frequency of CD4^{+} and CD8^{+} T cells in WT and *Ccl8-/-* mice (Q, left). Statistical analysis of the number of CD4^{+} (R) and CD8^{+} (S) T cells in WT and *Ccl8-/-* mice (right). Values represent mean ± SD from three independent experiments (n = 3 mice). *P* values were calculated by two-tailed unpaired Student's t-test.

T-V. Representative flow plots showing the frequency of NK and NKT cells in WT and *Ccl8-/-* mice (T). Statistical analysis of the number of NK (U) and NKT (V) cells in WT and *Ccl8-/-* mice. Values are mean ± SD from three independent experiments (n =

3 mice). *P* values were calculated by two-tailed unpaired Student's t-test.

W-X. Representative flow plots showing the frequency of $CD25^+$ $Foxp3^+$ cells in WT and *Ccl8-/-* mice (W). Statistical analysis of the number of $CD25^+$ $Foxp3^+$ cells in WT and *Ccl8-/-* mice (X). Values are mean ± SD from three independent experiments (n = 3 mice). *P* values were calculated by two-tailed unpaired Student's t-test.

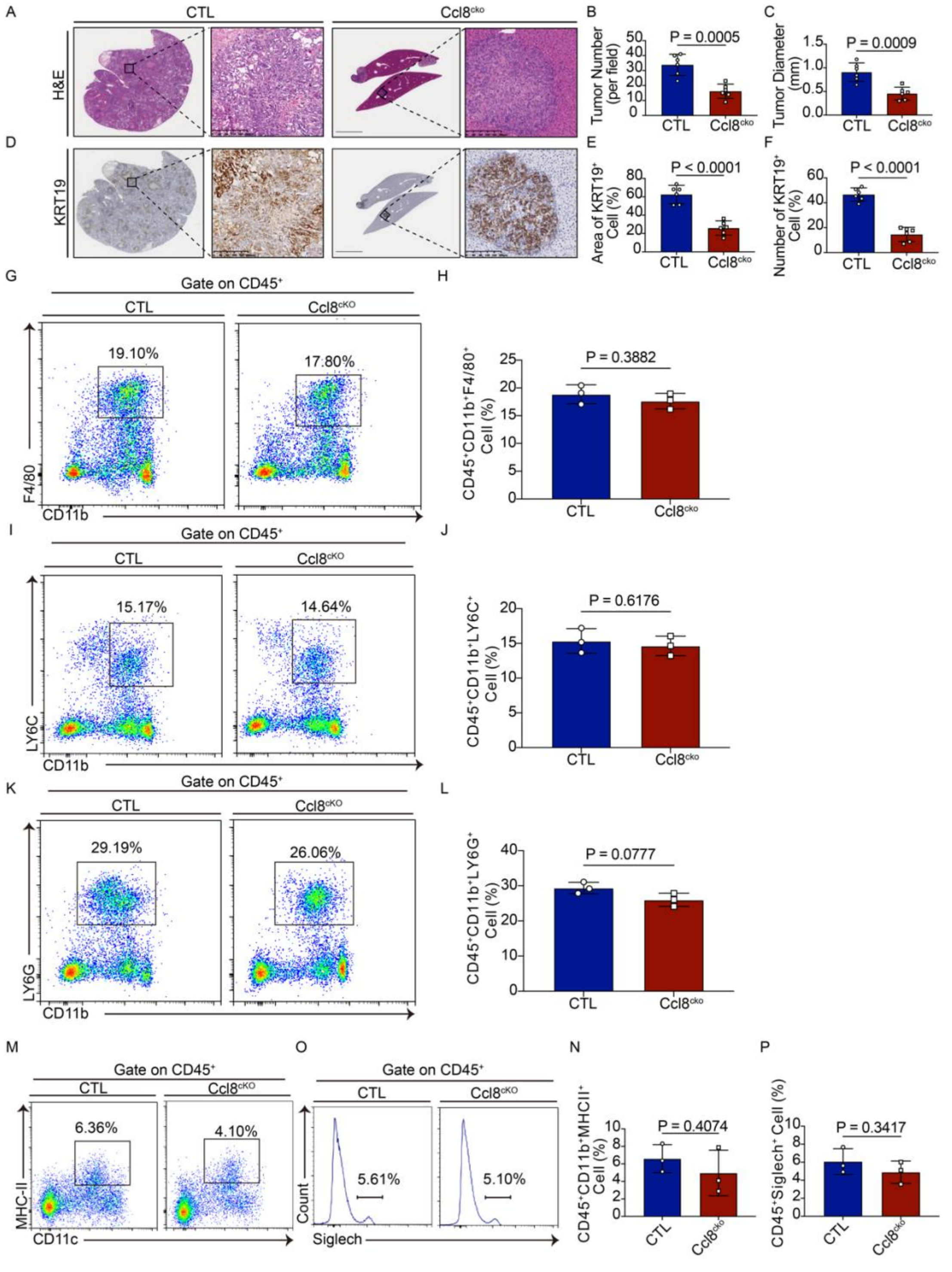

Figure S10 Related to Figure. CCL8 is the downstream mediator of AXL signaling in Reg-TAMs.

A-F. Representative images of H&E staining (A) and KRT19 staining (D) of liver sections in control (CTL) and *Lyz2-creER; Ccl8*$^{flox/flox}$ (Ccl8^{cKO}) groups, with scale bars of 200 μm. Statistical analyses include ICC number (B), ICC diameter (C), KRT19^{+}

cells (E), and KRT19$^+$ area (F) in CTL and Ccl8^{cKO} groups. Values represent mean ± SD from six independent biological replicates (n = 6 mice). *P* values were calculated by two-tailed unpaired Student's t-test.

G and H. Representative flow plots show the frequency of the CD11b$^+$ F4/80$^+$ macrophages as indicated in CTL and Ccl8^{cKO} groups (G). Quantification of the CD11b$^+$ F4/80$^+$ cells in CTL and Ccl8^{cKO} groups (H). Values are mean ± SD (n = 3 mice). *P* value was calculated by two-tailed unpaired Student's t-test.

I and J. Representative flow plots show the frequency of the CD11b$^+$ LY6C$^+$ monocytes as indicated in CTL and Ccl8^{cKO} groups (I). Quantification of the CD11b$^+$ LY6C$^+$ cells in CTL and Ccl8^{cKO} groups (J). Values are mean ± SD (n = 3 mice). *P* value was calculated by two-tailed unpaired Student's t-test.

K and L. Representative flow plots show the frequency of the CD11b$^+$ LY6G$^+$ neutrophils as indicated in CTL and Ccl8^{cKO} groups (K). Quantification of the CD11b$^+$ LY6G$^+$ cells in CTL and Ccl8^{cKO} groups (L). Values are mean ± SD (n = 3 mice). *P* value was calculated by two-tailed unpaired Student's t-test.

M and N. Representative flow plots show the frequency of the CD11c$^+$ MHC II$^+$ cDCs as indicated in CTL and Ccl8^{cKO} groups (M). Quantification of the CD11c$^+$ MHC II$^+$ cells in CTL and Ccl8^{cKO} groups (N). Values are mean ± SD (n = 3 mice). *P* value was calculated by two-tailed unpaired Student's t-test.

O and P. Representative histogram plots show the frequency of the Siglech$^+$ pDCs as indicated in CTL and Ccl8^{cKO} groups (O). Quantification of the Siglech$^+$ cells in CTL and Ccl8^{cKO} groups (P). Values are mean ± SD (n = 3 mice). *P* value was calculated by two-tailed unpaired Student's t-test.

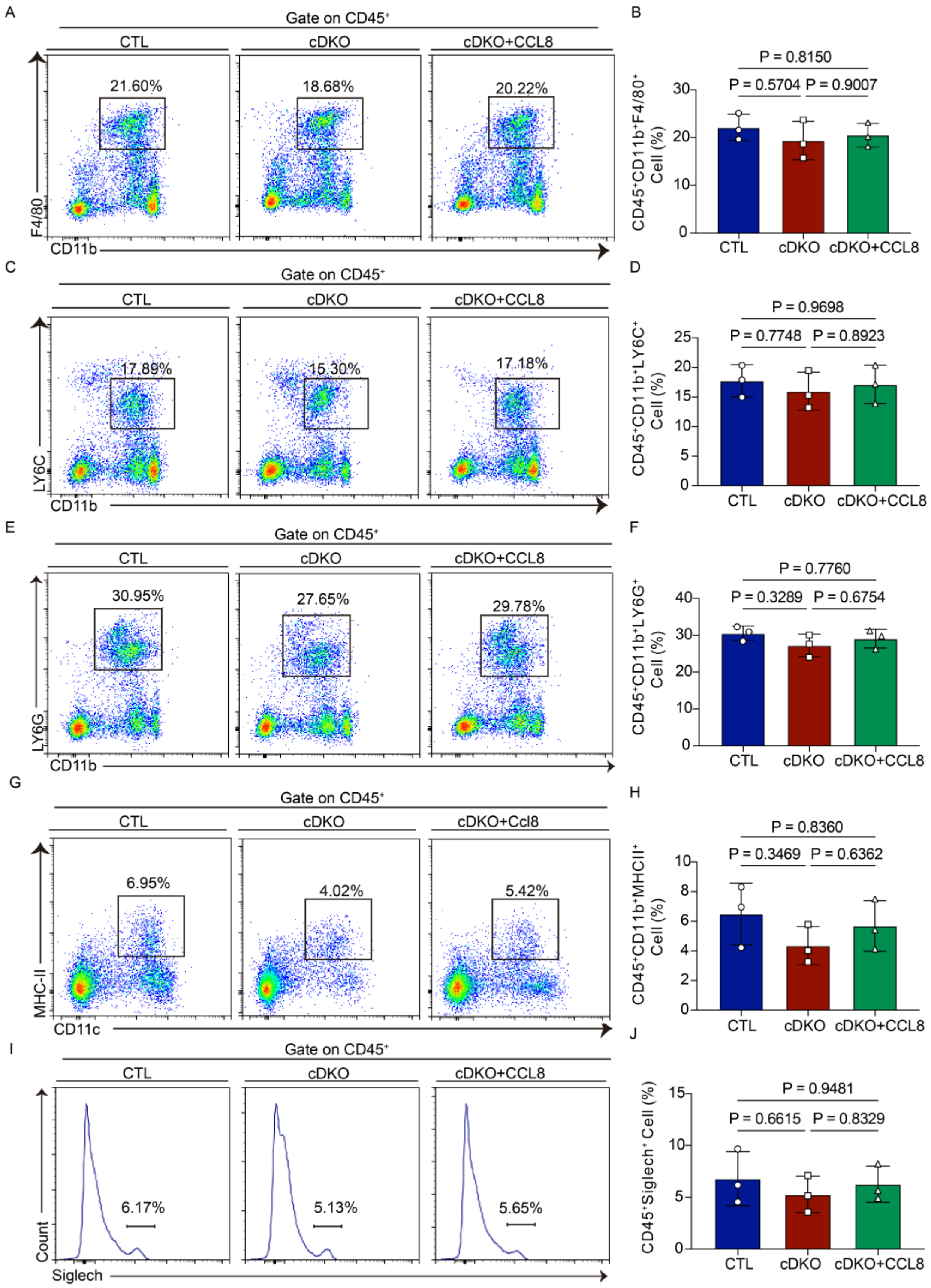

Figure S11 Related to Figure. CCL8 is the downstream mediator of AXL signaling in Reg-TAMs

A and B. Representative flow plots show the frequency of the CD11b$^+$ F4/80$^+$ macrophages as indicated in control (CTL), *Lyz2-creER; Axl$^{flox/flox}$; Mertk$^{flox/flox}$* (Double

Knockout, cDKO group) and *Lyz2-creER; Axl$^{flox/flox}$; Mertk$^{flox/flox}$* +CCL8 (cDKO+CCL8) groups (A). Quantification of the CD11b$^+$ F4/80$^+$ cells in CTL, cDKO, and cDKO+CCL8 groups (B). Values are mean ± SD (n = 3 mice). *P* values were calculated by one-way ANOVA with Tukey's multiple comparison test.

C and D. Representative flow plots show the frequency of the CD11b$^+$ LY6C$^+$ monocytes as indicated in CTL, cDKO, and cDKO+CCL8 groups (C). Quantification of the CD11b$^+$ LY6C$^+$ cells in CTL, cDKO, and cDKO+CCL8 groups (D). Values are mean ± SD (n = 3 mice). *P* values were calculated by one-way ANOVA with Tukey's multiple comparison test.

E and F. Representative flow plots show the frequency of the CD11b$^+$ LY6G$^+$ neutrophils as indicated in CTL, cDKO, and cDKO+CCL8 groups (E). Quantification of the CD11b$^+$ LY6G$^+$ cells in CTL, cDKO, and cDKO+CCL8 groups (F). Values are mean ± SD (n = 3 mice). *P* values were calculated by one-way ANOVA with Tukey's multiple comparison test.

G and H. Representative flow plots show the frequency of the CD11c$^+$ MHC II$^+$ cDCs as indicated in CTL, cDKO, and cDKO+CCL8 groups (G). Quantification of the CD11c$^+$ MHC II$^+$ cells in CTL, cDKO, and cDKO+CCL8 groups (H). Values are mean ± SD (n = 3 mice). *P* values were calculated by one-way ANOVA with Tukey's multiple comparison test.

I and J. Representative histogram plots show the frequency of the Siglech$^+$ pDCs as indicated in CTL, cDKO, and cDKO+CCL8 groups (I). Quantification of the Siglech$^+$ cells in CTL, cDKO, and cDKO+CCL8 groups (J). Values are mean ± SD (n = 3 mice). *P* values were calculated by one-way ANOVA with Tukey's multiple comparison test.

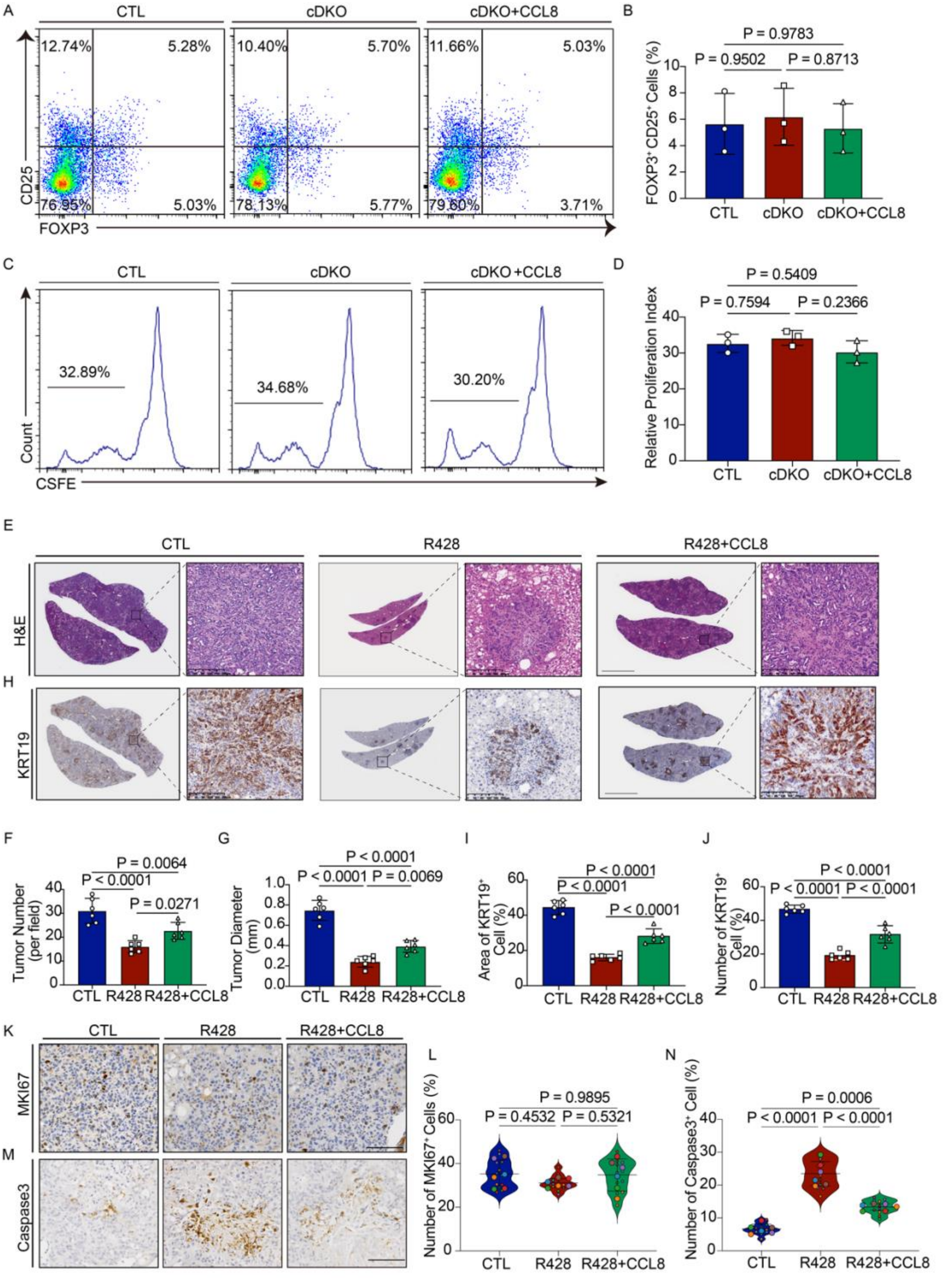

Figure S12 Related to Figure. CCL8 is the downstream mediator of AXL signaling in Reg-TAMs

A and B. Representative flow plots (A) and corresponding graph of the frequency of $CD25^{+}Foxp3^{+}$ Treg cells in control (CTL), *Lyz2-creER; Axl*$^{flox/flox}$*; Mertk*$^{flox/flox}$ (*Axl* and *Mertk* conditional double knockout, cDKO group) and *Lyz2-creER; Axl*$^{flox/flox}$*;*

Mertk$^{flox/flox}$ +CCL8 (cDKO+CCL8) groups(B). Values are mean ± SD from three independent experiments (n = 3 mice). *P* values were calculated by one-way ANOVA with Tukey's multiple comparison test.

C and D. Representative histograms show CFSE intensity of $CD3^+CD8^+$ T cells (C) and the corresponding relative proliferation index (D) in CTL, cDKO, and cDKO+CCL8 groups, as assessed by flow cytometry. Values are mean ± SD from three independent experiments (n = 3 mice), and *P* values were calculated by one-way ANOVA with Tukey's multiple comparison test.

E-J. Representative images of H&E staining (E) and KRT19 staining (H) of liver sections in different treatment groups, with scale bars of 200 μm. Statistical analyses include ICC number (F), ICC diameter (G), $KRT19^+$ cells (I), and $KRT19^+$ area (J) across different treatment groups. Values represent mean ± SD from six independent biological replicates (n = 6 mice). *P* values were calculated by one-way ANOVA with Tukey's multiple comparison test.

K and M. Representative images of MKI67 (K) and active-Caspase3 (M) staining of liver sections in different treatment groups. Scale bar, 100μm.

L and N. Statistical analysis of $MKI67^+$ (L) and active-$Caspase3^+$ cells (N) in different treatment groups. Values are mean ± SD from six independent biological replicates (n = 12 fields from 6 mice). *P* values were calculated by one-way ANOVA with Tukey's multiple comparison test.

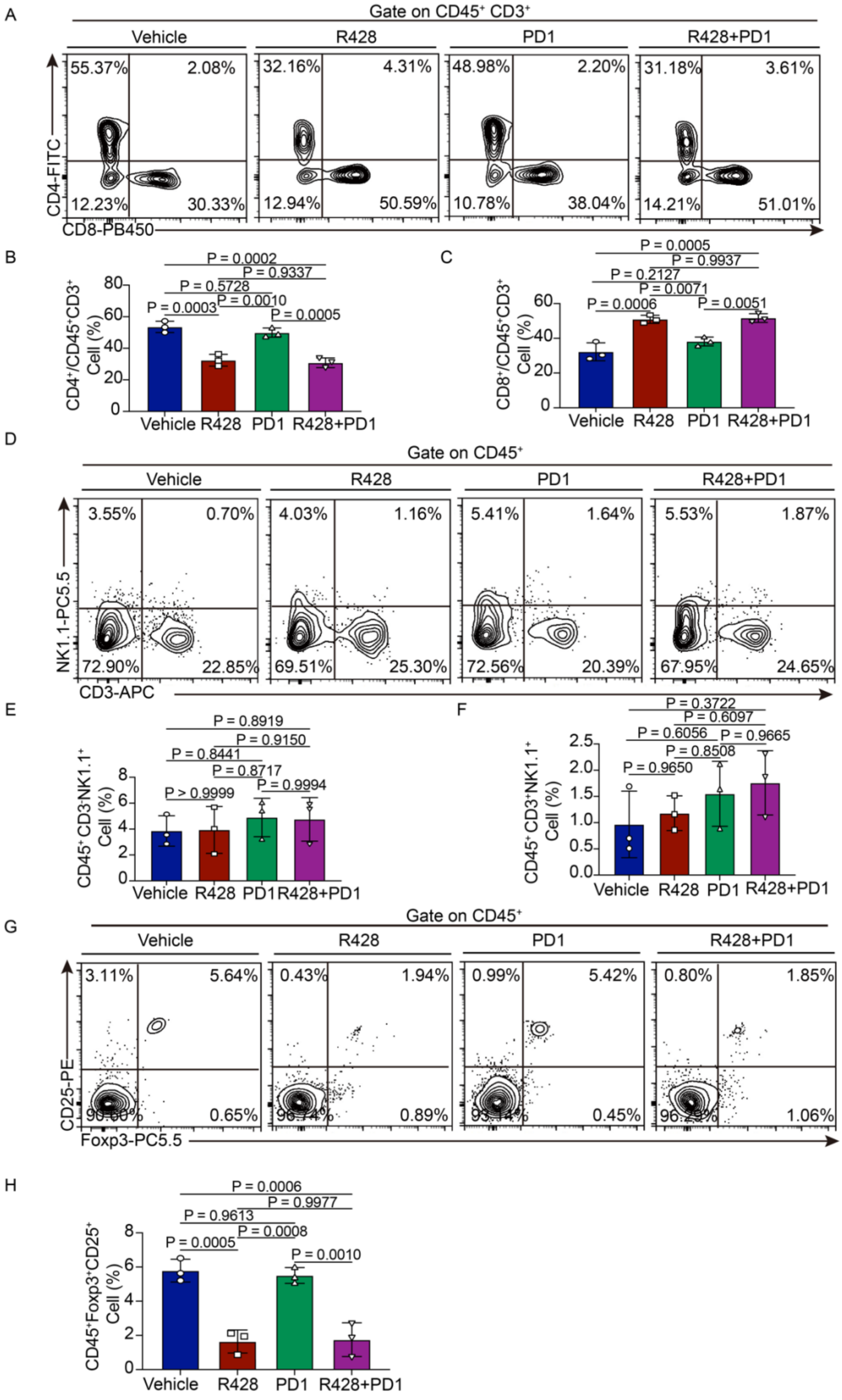

Figure S13. Related to Figure 7. R428 treatment sensitizes murine ICC cells to anti-

PD-1 treatment

A-C. Representative flow plots (A) and corresponding graphs of the frequency of tumor-infiltrating $CD4^+$ (B), and $CD8^+$ (C) T cells in different treatment groups. Error bars are mean ± SD from three independent experiments (n = 3 mice). *P* values were calculated by one-way ANOVA with Tukey's multiple comparison test.

D-F. Representative flow plots (D) and corresponding graphs of the frequency of tumor-infiltrating NK cells (E) and tumor-infiltrating NKT cells (F) in different treatment groups. Error bars are mean ± SD from three independent experiments (n = 3 mice). *P* values were calculated by one-way ANOVA with Tukey's multiple comparison test.

G and H. Representative flow plots (G) and corresponding graph of the frequency of tumor-infiltrating Treg cells (H) in different treatment groups. Values are mean ± SD from three independent experiments (n = 3 mice). *P* values were calculated by one-way ANOVA with Tukey's multiple comparison test.

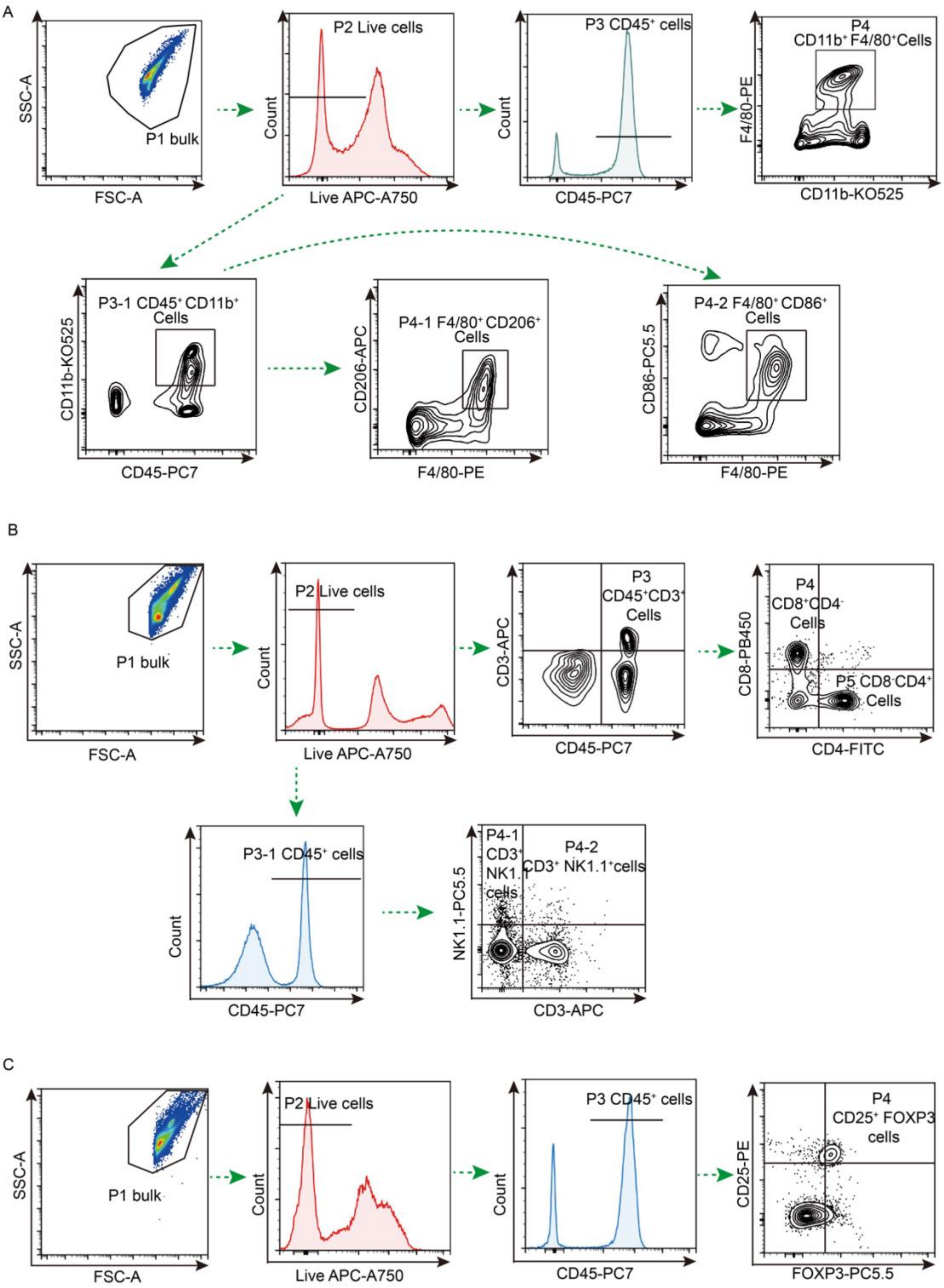

Figure S14. Flow cytometry gating strategies

A-C. Gating strategies were used in this study to define different macrophage populations (A), T cell populations (B), and Tregs (C).
