# Supplementary figures and images for: Tumor-initiating cells escape tumor immunity via CCL8 from tumor-associated macrophages in mice
Source: J Clin Invest. 2025 Jan 7;135(5):e180893. doi: 10.1172/JCI180893 (PMC11870738; doi:10.1172/JCI180893)

Figure 5M

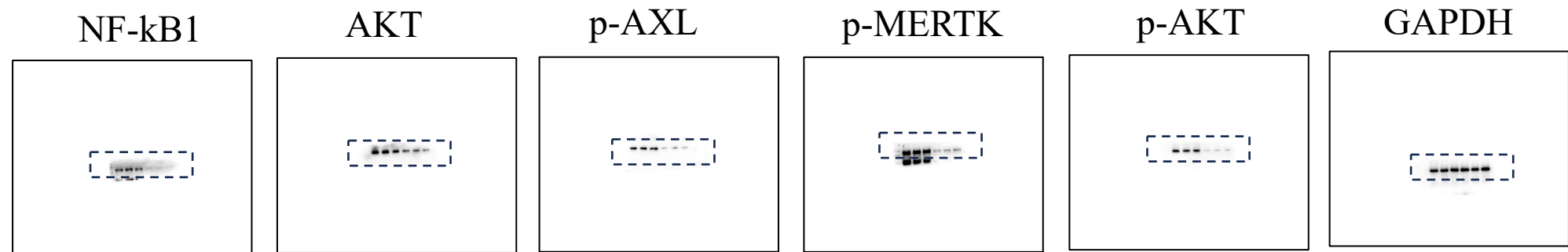

Figure 6B

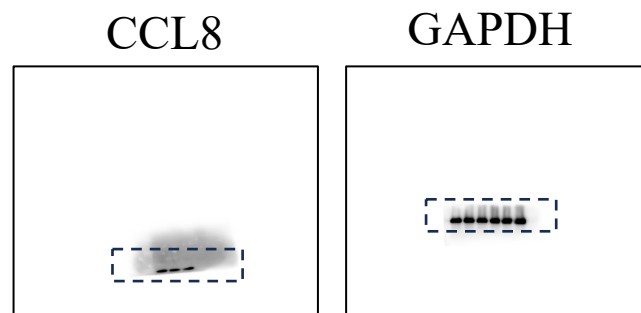

Figure S3J

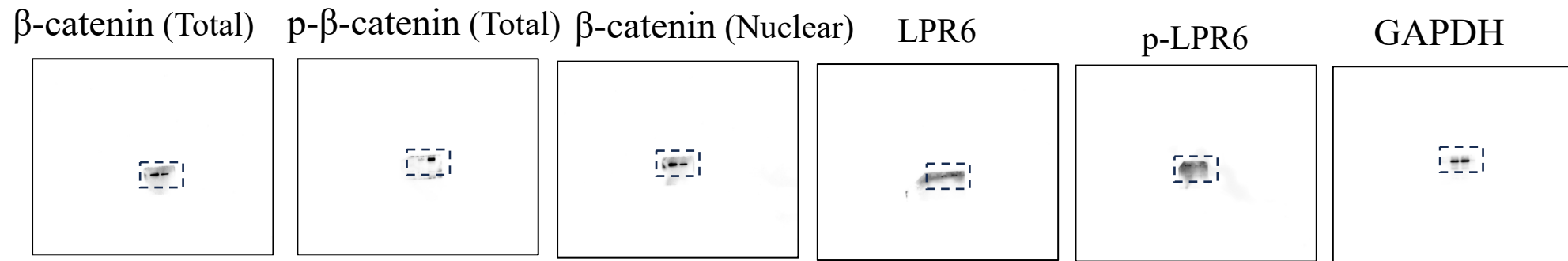

Figure S6I

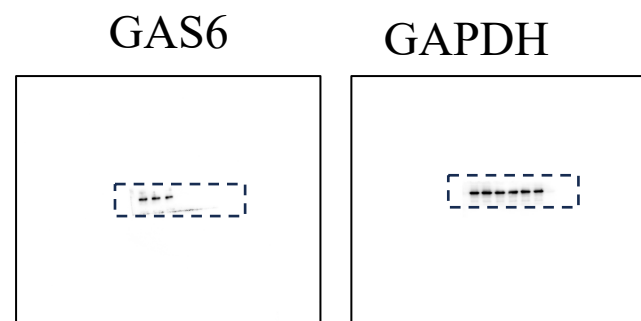

Figure S6S

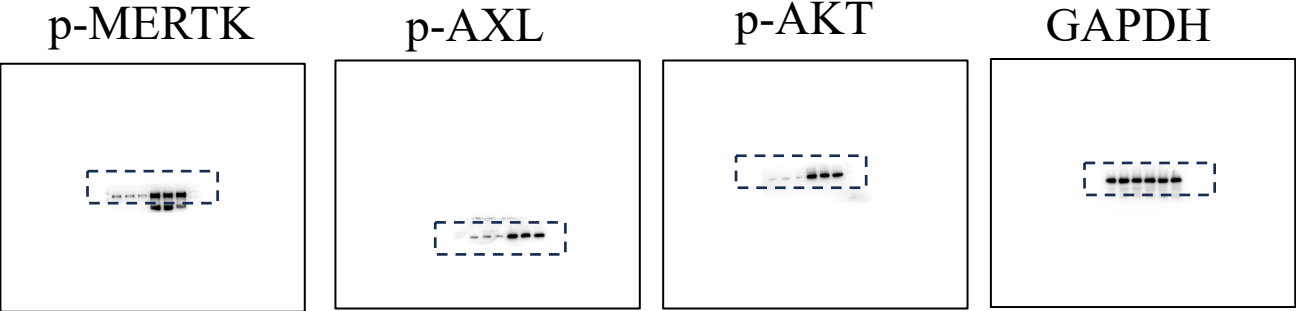

Figure S7K

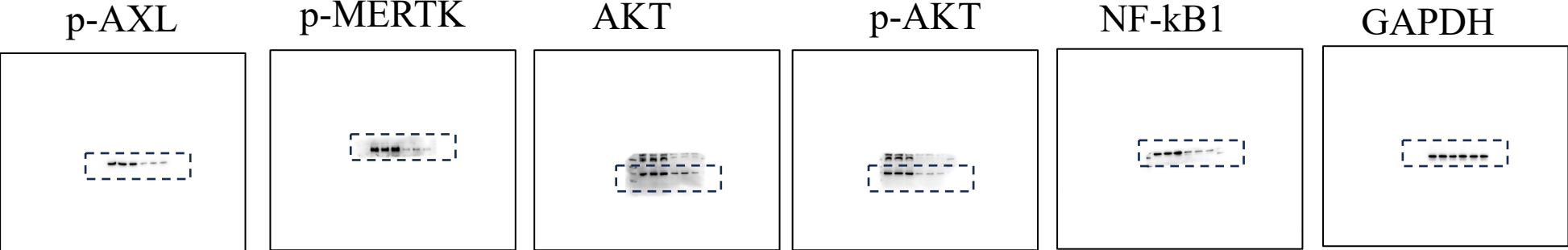

Figure S8A

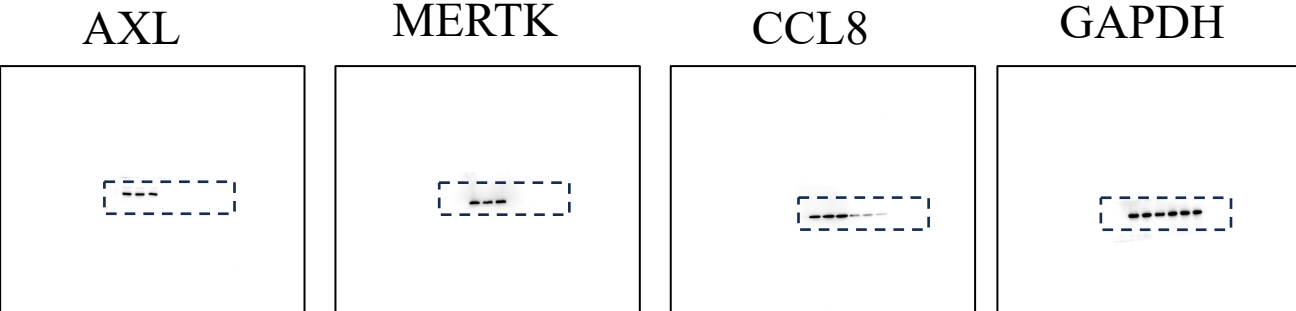

Figure S9D

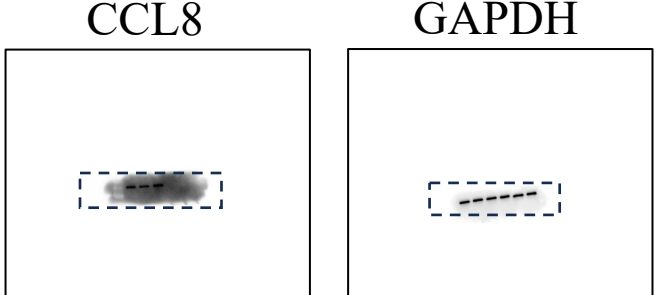

Supplement: Unedited blot and gel images [file jci-135-180893-s009.pdf]
